# Effectiveness and gender-tailoring of suicide prevention interventions for men: a systematic review

**DOI:** 10.1186/s12889-026-28269-1

**Published:** 2026-06-23

**Authors:** Lan Shi, Sara Sutori, Ulrika Lögdberg, Vladimir Carli, Emma Therése Eliasson

**Affiliations:** Department of Learning, Informatics, Management and Ethics, National Centre for Suicide Research and Prevention, Karolinska Institutet, Stockholm, Sweden

**Keywords:** Suicide prevention, Men, Gender-tailoring, Masculinity, Stigma, Help-seeking, Systematic review

## Abstract

**Background:**

Men have 2–4 times higher suicide mortality than women and are less likely to seek help, with disparities linked to restrictive masculine norms and stigma. This review aimed to synthesize the effectiveness of suicide prevention interventions targeting men and identify gender-tailoring strategies.

**Methods:**

Studies evaluating suicide prevention interventions targeting men were included if conducted in male-only samples and reporting outcomes related to suicidality (e.g., suicide deaths, suicide attempts, suicidal ideation), depression, or help-seeking (e.g., behaviors, intentions, attitudes). Six databases (MEDLINE, Embase, PsycINFO, Cochrane Library, Web of Science, and CINAHL) were searched for peer-reviewed English-language studies published up to 31 December 2025. Risk of bias was assessed using RoB 2 and ROBINS-I. Due to heterogeneity, findings were synthesized narratively. The protocol was registered in PROSPERO (CRD420250655554).

**Results:**

Seventeen articles evaluating 14 interventions were included. Only one study assessed suicide deaths, and none occurred. Among RCTs, no significant effects were observed for suicide attempts, suicidal ideation, or depression. Help-seeking behaviors and intentions showed mixed evidence of improvement, with no improvement in help-seeking attitudes. Gender-tailoring strategies were synthesized into seven categories: intervention design and development, risk targeting, content and messaging, outreach and recruitment, delivery modalities, male representation and role modeling, and communication and action orientation.

**Conclusions:**

Evidence for the effectiveness of suicide prevention interventions targeting men remains limited, with no clear effects on suicidality or depression and mixed evidence for improvements in help-seeking. Risk of bias was generally moderate to high. A wide range of gender-tailoring strategies was identified. Further development of gender-responsive approaches and more rigorous evaluation are needed to reduce male suicide mortality.

**Supplementary Information:**

The online version contains supplementary material available at https://doi.org/10.1186/s12889-026-28269-1.

## Introduction

Suicide is a significant global public health challenge and accounts for over 720,000 deaths annually worldwide [1]. Men are disproportionately affected: although women report higher rates of suicidal ideation and non-fatal suicide attempts, men are two to four times more likely to die by suicide [2–4], a pattern commonly referred to as the gender paradox in suicidal behavior [5]. This excess male mortality has substantial social and economic consequences, contributing to premature mortality and productivity losses [6, 7]. Established risk factors for suicide include depression, prior suicide attempts or self-harm, substance misuse, and social or relationship stressors [8, 9]. However, men face additional vulnerabilities that may contribute to their higher suicide mortality, including greater use of highly lethal means [10, 11] and lower levels of help-seeking for psychological distress or suicidal thoughts [12].

Masculine norms and stigma play a central role in shaping male-specific barriers to help-seeking and mental health [13], thereby contributing to elevated suicide risk among men [14]. Traditional masculine norms emphasize stoicism, self-reliance, and toughness, promoting expectations of invulnerability [15]. Under these norms, men may be discouraged from expressing emotions, talking openly about problems, or seeking help, reflecting their restrictive influence [15, 16]. Such behaviors are often perceived as signs of weakness, inadequacy, or failure, as “unmanly” or “feminine”, and as incompatible with masculine ideals, thereby posing a threat to masculine identity [16, 17]. In addition, stigma in both public and internalized forms fosters fear of judgment and shame that inhibit emotional and communicative openness and help-seeking [16, 18]. As a result, psychological distress may accumulate over time. Consistent with this, depression in men may manifest in externalizing or “masked” forms, characterized by anger and irritability, self-destructive behaviors, and maladaptive coping strategies including risk-taking, substance use, and violence, rather than overt sadness or help-seeking [16, 19]. In severe cases, these dynamics may increase suicide risk, particularly as men are more likely to use immediately lethal and violent methods, which may be perceived as more masculine and powerful, potentially contributing to fatal outcomes [20]. Together, these processes describe a psychocultural pathway linking restrictive masculine norms and stigma to reduced help-seeking, poorer mental health, and elevated suicide risk among men.

Many mixed-gender or gender-neutral suicide prevention interventions may not explicitly address male-specific pathways to suicide risk. For example, many rely primarily on talking-based therapeutic approaches that emphasize emotional expression, which may conflict with restrictive masculine norms and be less appealing to some men [21]. Consequently, men are consistently less likely than women to participate in or complete suicide prevention interventions, which may contribute to lower observed effectiveness among men [22].

Given men’s lower engagement with gender-neutral suicide prevention interventions and the reduced effectiveness of these interventions for men, there is a need for suicide prevention interventions tailored specifically to men, while acknowledging that gender extends beyond binary categories [23]. In response, recent years have seen a growing number and diversity of male-focused suicide prevention initiatives, as documented in two scoping reviews [24, 25]. Alongside this development, researchers and practitioners have increasingly explored strategies for providing gender-sensitive support to men. A male-centered approach has been outlined as one that consciously considers men’s needs and preferences in the design, delivery, promotion, and continuous improvement of programs and services [26]. Proposed strategies include reframing help-seeking by redefining what it means to “be a real man”, consulting with the target group, recruiting men by “going to where men are”, delivering interventions in familiar, safe, and non-medical settings, and using language familiar to men [24, 27, 28].

To date, however, no systematic review has evaluated the effectiveness of suicide prevention interventions delivered exclusively to male populations. Existing reviews in this field have primarily relied on scoping approaches, without formal quality appraisal or systematic synthesis of intervention effectiveness [24, 25]. In addition, previous work was not restricted to male-only samples, as mixed-gender studies were included when results for men were reported separately [24].

In addition, while male-focused suicide prevention strategies have increasingly been proposed in the literature [24, 27, 28], these strategies are typically presented as recommendations. No study has systematically examined how gender-tailoring strategies are practically integrated in structured, real-world suicide prevention interventions for men.

To address these gaps, this systematic review aims to synthesize evidence on suicide prevention interventions targeting men. Specifically, it addresses two research questions: (1) What is the effectiveness of suicide prevention interventions targeting men in reducing suicide-related outcomes, reducing depression, and improving help-seeking outcomes? (2) What gender-tailoring strategies do suicide prevention interventions incorporate to address male-specific barriers to help-seeking and mental health?

## Methods

This systematic review followed the PRISMA 2020 checklist [29]. The review protocol was prospectively registered in PROSPERO (CRD420250655554) [30].

### Inclusion criteria

This review included studies evaluating the effectiveness of suicide prevention interventions targeting men, designed to reduce or prevent suicidality and conducted in male-only samples. Eligible studies were required to report at least one quantitative outcome in one of the following domains: (a) suicide-related outcomes (e.g., suicide deaths, suicide attempts, or suicidal ideation); (b) depression; or (c) help-seeking outcomes (e.g., behaviors, intentions, or attitudes), conceptually informed by the Theory of Planned Behavior [31]. Eligible study designs included randomized controlled trials (RCTs), quasi-experimental studies, cohort studies, case-control studies, and (repeated) cross-sectional studies. Mixed-methods studies were included when the quantitative component met these criteria. Comparators could include any control condition or no control group. No restrictions were placed on study setting or context.

### Exclusion criteria

Studies were excluded if the intervention was not explicitly aimed at reducing or preventing suicidality (e.g., general mental health interventions that assessed suicide-related outcomes), or if medication was the only intervention component. Regarding population, studies were excluded if they were mixed-gender studies, focused on women/females or other genders/sexes, or targeted indirect populations such as gatekeepers, practitioners, or crisis supporters. Regarding outcomes, studies assessing only implementation outcomes or process evaluations (e.g., feasibility or acceptability), as well as studies lacking quantitative outcome data, were excluded. Regarding study design, qualitative studies (including qualitative components of mixed-methods studies), ecological studies, case reports, case series, and reviews were excluded. In addition, studies that did not evaluate intervention effectiveness (e.g., epidemiological studies of risk factors or mediation analyses) were excluded.

### Information sources, search strategy, and study selection

The search strategy was developed in collaboration with librarians at Karolinska Institutet. Peer-reviewed original research studies were retrieved from six databases: MEDLINE (Ovid), Embase, PsycINFO, the Cochrane Library (including CENTRAL), Web of Science, and CINAHL. Inclusion was restricted to studies published in English to support consistent screening, data extraction, and risk of bias assessment across the multinational review team, for whom English was the shared working language. Searches combined controlled vocabulary (e.g., MeSH terms) with free-text keywords related to suicide, men, and interventions. The initial search covered studies published up to February 28, 2025, with an updated search including studies published up to December 31, 2025. Reference lists of included studies and relevant reviews were screened to identify additional records. Study protocols, conference abstracts, and grey literature were excluded. Study selection was conducted in two stages: title and abstract screening followed by full-text screening. All screening decisions were recorded in Covidence, and the selection process is reported using a PRISMA 2020 flow diagram. Full search strategies are available in the preregistration materials [30].

### Data extraction

Data were extracted from published papers, trial registries, study protocols, and supplementary materials of the included studies. Study authors were contacted when clarification or additional information was required.

Extracted study characteristics included first author, publication year, country, setting, study design, sample size, and funding sources.

Participant characteristics included age range, demographic or subpopulation characteristics, and baseline levels of suicide risk, depression, or other mental health risk.

Intervention characteristics included intervention name, core components and content, delivery mode and facilitation, structure and intensity (e.g., total number of sessions, session length, frequency, and timing), intervention duration, and follow-up period. Definitions of the intervention/exposure group or condition and the comparison group or condition were extracted, along with the type of control condition (active or passive).

Effectiveness outcomes were organized into three main domains: (a) suicide-related outcomes, (b) depression, and (c) help-seeking outcomes. For each domain, data were extracted for relevant sub-outcomes. Extracted information included specific outcome measures, assessment timepoints, and whether results reflected between-group or within-group/pre–post comparisons. Effect estimates were extracted together with corresponding confidence intervals and p-values, and summary statistics where available.

For this review, gender-tailoring for men was defined as any intentional adaptation of intervention content, design, delivery, or implementation to address male-specific needs, preferences, or norms. Gender-tailoring data were extracted from intervention descriptions where authors explicitly described the intervention as tailored to men, provided a male-specific rationale, or referenced relevant literature on masculinity, male help-seeking, mental health, depression, or suicide risk. Publicly available intervention materials (e.g., websites or videos) were also reviewed.

### Risk of bias assessment

Risk of bias was assessed using the revised Cochrane risk-of-bias tool for randomized trials (RoB 2) and the Risk Of Bias In Non-randomized Studies – of Interventions, Version 2 (ROBINS-I V2, 2024), in accordance with the Cochrane Handbook for Systematic Reviews of Interventions [32–34].

### Data synthesis

To synthesize findings regarding the effectiveness of male suicide prevention interventions (research question 1), a narrative synthesis was conducted, given anticipated heterogeneity in outcome measures, assessment timepoints, effect measures, and analytical approaches. Findings were categorized into three outcome domains (suicide-related outcomes, depression, and help-seeking outcomes) and further organized by identical or conceptually similar sub-outcomes. Evidence was considered in relation to study design and risk of bias. For controlled studies, effectiveness was assessed primarily using between-group comparisons, whereas for uncontrolled studies, within-group or pre–post changes were summarized to provide contextual information.

To synthesize findings regarding gender-tailoring strategies (research question 2), a second narrative synthesis was conducted. In the absence of an established framework for male-specific suicide prevention strategies, an exploratory approach was adopted. Gender-tailoring elements were identified across interventions through iterative comparison of recurring features and practices and were subsequently grouped into higher-order categories representing common gender-tailoring strategies.

### Review process

Title and abstract screening and full-text screening were conducted independently by two reviewers. Disagreements during screening were resolved by a third reviewer, who was blinded to the initial decisions. Data extraction was conducted by one reviewer and verified by a second reviewer. Risk of bias assessment was performed independently by two reviewers. Synthesis of effectiveness outcomes (research question 1) was conducted by one reviewer and verified by a second reviewer. Synthesis of gender-tailoring strategies (research question 2) was conducted independently by two reviewers. Disagreements during data extraction, risk of bias assessment, and data synthesis were resolved through discussion and consensus; if necessary, a third reviewer was consulted.

## Results

### Study selection

**Fig. 1:**
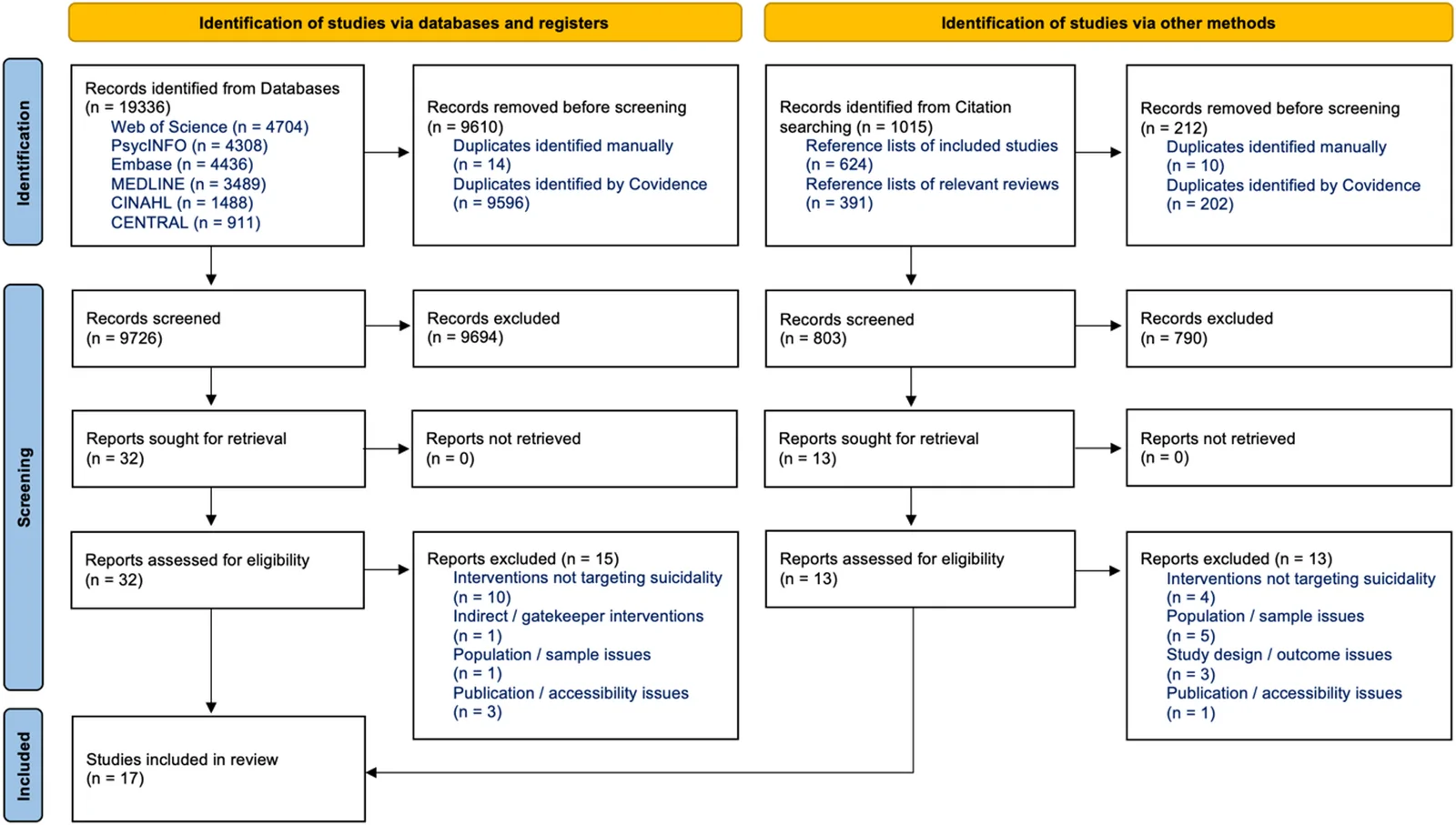
PRISMA 2020 flow diagram of study selection

Based on database searches covering records published up to December 31, 2025, a total of 19,336 records were identified for screening. After deduplication, 9,726 titles and abstracts were screened, followed by full-text assessment of 32 articles. Ultimately, 17 articles met the inclusion criteria. Screening of the reference lists of included articles and relevant reviews identified no additional eligible articles. The study selection process is presented in the PRISMA flow diagram (Fig. 1). A list of articles excluded after full-text screening, with reasons for exclusion, is provided in Supplementary Table S1.

### Study characteristics

The publication years of the 17 included articles ranged from 2006 to 2025, with 14 articles published in or after 2015, indicating that the evidence base has expanded over the past decade (Table 1). Regarding study design, the majority were RCTs (*n* = 7 studies), followed by one non-randomized controlled trial (NRCT), two controlled cohort studies, two one-group pretest–posttest studies, one repeated cross-sectional study, one cross-sectional study, and one multi-design study combining pre–post and cross-sectional elements. In some cases, multiple articles reported findings from the same study (e.g., secondary analyses or reports of different outcomes). Across the 17 articles, 14 distinct interventions were evaluated. All interventions were conducted in high-income countries: five in Australia, three in Canada, two in the United States, two in the United Kingdom, one in Belgium, and one in Japan. Interventions were delivered across diverse settings, including media-based (*n* = 5), community-based (*n* = 3), combined media- and community-based (*n* = 1), and mobile-based (*n* = 1). Other settings included prisons, schools, workplaces, and primary care clinics.

**Table 1:** Key characteristics of included articles

First author	Publication year	Country	Intervention name	Setting	Study design	Sample size	Type of control condition
Frey [35]	2022	U.S.	*Man Therapy*	media-based	RCT (primary analysis)	Int 171 Con 206	active control
Gilgoff [36]	2022	U.S.	*Man Therapy*	media-based	RCT (secondary analysis)	Int 162 Con 192	active control
Stas [37]	2023	Belgium	*Get out of your head*	media-based	one-group pretest–posttest design	Pre 203 Post 115	n/a
Ogrodniczuk [38]	2024	Canada	*HeadsUpGuys*	media-based	cross-sectional study	443	n/a
King [39]	2017	Australia	*Man Up*	media-based	RCT	Int 169 Con 168	active control
Schlichthorst [40]	2018	Australia	*Man Up*	media-based	repeated cross-sectional study	Pre 476 Post-Exp 619 Post-UnExp 192	passive control (no exposure)
Nicholas [41]	2025	Australia	*Boys Do Cry*	media-based	RCT	Int 243 Con 233	active control
Daigle [42]	2006	Canada	*Suicide Prevention Week 2000* / *SPW 2000*	media- and community-based	combined pre–post and cross-sectional design	Exp 190 UnExp 830	passive control (no exposure)
Milner [43]	2019	Australia	*Contact+Connect*	mobile-based	RCT (different outcomes)	Int 227 Con 215	passive control (waitlist)
Milner [44]	2017	Australia	*Contact+Connect*	mobile-based	RCT (different outcomes)	Int 247 Con 231	passive control (waitlist)
Jerant [45]	2020	U.S.	*Men and Providers Preventing Suicide* / *MAPS*	primary care clinic-based	RCT	Int 21 Con 27	active control
Nakao [46]	2007	Japan	*Employee Assistance Programme* / *EAP*	workplace-based	cohort study	Exp 283 UnExp 22	active control
Calear [47]	2021	Australia	*Silence is Deadly*	school-based	NRCT	Int 418 Con 176	passive control (waitlist)
Pratt [48]	2015	U.K.	*Cognitive Behavioural Suicide Prevention* / *CBSP*	prison-based	RCT	Int 31 Con 31	active control
Heisel [49]	2019	Canada	*Meaning-Centered Men’s Groups* / *MCMG*	community-based	one-group pretest–posttest design	Pre 27 Post 27	n/a
Jackson [50]	2022	U.K.	*Hope service*	community-based	cohort study within mixed-methods design	M/H Exp 64 L Exp 16	active control
De Leo [51]	2007	Australia	*Intensive Case Management* / *ICM*	community-based	RCT	Int 14 Con 8	active control

*Abbreviations: Int* intervention group, *Con* control group, *Pre* pre-assessment, *Post* post-assessment, *Exp* exposed group, *UnExp* unexposed group, *M/H Exp* moderate/high-intensity exposure, *L Exp* low-intensity exposure, *n/a* not applicable

Across the included studies, participant ages ranged from 16 to 87 years (Supplementary Table S2). Most interventions focused on adult and working-aged men (typically 25–64 years), while two studies targeted younger or older participants (adolescents and men facing retirement). Studies recruited either general male population samples or specific groups of men considered vulnerable, such as construction workers, prisoners, men experiencing financial difficulties, and men following discharge from inpatient psychiatric care. Baseline suicide and depression risk varied across studies, ranging from general population levels to elevated clinical risk, with some studies excluding men at active suicide risk. Table 1 presents the key characteristics of the included articles. Detailed participant characteristics, intervention and comparator conditions, and funding sources are provided in Supplementary Table S2.

### Overview of interventions

Fourteen distinct interventions were identified across the included studies. Six interventions were media- and public awareness-focused campaigns, five of which were primarily delivered digitally. *Man Therapy*, *Get out of your head*, and *HeadsUpGuys* were websites, while *Man Up* was a documentary and *Boys Do Cry* was a music video. *Man Therapy* [35, 36] integrated humor, male-oriented language and metaphors, and a fictional male host to reduce stigma and reframe help-seeking as a sign of strength. *Get out of your head* [37] used campaign videos, testimonials, and mental health and suicide prevention information to reduce stigma and promote recognition of suicidality, help-seeking, and peer support. *HeadsUpGuys* [38] offered strategies for managing and recovering from depression and suicidality and promoted a strength-based, graduated pathway from self-management to peer and professional help-seeking. *Man Up* [39, 40] explored links between masculinity, help-seeking, men’s mental health, and suicidality, incorporating behavioral modeling, personal stories, and expert insights. *Boys Do Cry* [41] encouraged men to express their feelings and seek support when experiencing mental health difficulties, with the call to action “When the going gets tough. Get Talking”. Additionally, *Suicide Prevention Week 2000* (*SPW 2000*) [42] combined media campaigns with community activities, challenging beliefs that men must not feel pain or express suffering and promoting help-seeking.

Two further interventions were delivered using digital technologies. *Contact+Connect* [43, 44] was a brief contact intervention for construction workers, delivered via smartphone text messages to reduce mental health stigma and promote long-term contact. *Men and Providers Preventing Suicide* (*MAPS*) [45] was a tailored interactive computer program designed to encourage disclosure and discussion of recent suicidal thoughts during subsequent primary care visits.

Workplace- and school-based initiatives were also identified. *Employee Assistance Programme* (*EAP*) [46] was a workplace support service offering anonymous psychological counseling, psychiatric referrals, and job-related mental health seminars. *Silence is Deadly* [47] primarily featured a school-based psychoeducational presentation for adolescent boys, using male role modeling and social norming to challenge masculine norms and promote early help-seeking and peer support.

Two interventions involved structured group psychotherapy. *Cognitive Behavioural Suicide Prevention* (*CBSP*) [48] offered cognitive-behavioral therapy to prevent suicide and was delivered to male prisoners. *Meaning-Centered Men’s Groups* (*MCMG*) [49] offered meaning-centered therapy to enhance meaning in life and psychological resilience among men struggling with the transition to retirement.

Finally, crisis and recovery-oriented interventions were also identified. *Hope service* [50] used face-to-face motivational interviewing to address acute distress and financial difficulties, combined with phone calls and text messages. *Intensive Case Management* (*ICM*) [51] was a post-discharge program for men following inpatient psychiatric care, providing face-to-face case management and outreach telephone check-ins.

Collectively, the interventions spanned universal, selective, and indicated approaches, reflecting the public health prevention continuum and ranging from broad population-level strategies to more targeted and intensive support. Detailed intervention characteristics are provided in Supplementary Table S2.

### Risk of bias assessment

Risk of bias assessments for each article and domain are presented in Table 2, with detailed justifications provided in Supplementary Tables S3 and S4. Among the nine randomized articles included, two were judged at overall low risk of bias, four at some concerns, and three at high risk of bias. The most common concerns in randomized articles related to missing outcome data, deviations from intended interventions, and selection of the reported result. Among the eight non-randomized articles included, one was judged at moderate risk of bias and seven at serious risk of bias. The most common concerns in non-randomized articles were confounding and missing outcome data.

**Table 2:** Risk of bias assessment of included articles

Randomized articles	Randomization process		Deviations from intended interventions		Missing outcome data		Measurement of outcome		Selection of reported result		Overall	
Frey [35]	low		high		high		some concerns		some concerns		high	
Gilgoff [36]	low		high		high		some concerns		high		high	
King [39]	low		low		low		some concerns		low		low	
Nicholas [41]	low		low		low		some concerns		low		low	
Milner [43]	low		some concerns		high		some concerns		some concerns		some concerns	
Milner [44]	low		some concerns		high		some concerns		some concerns		some concerns	
Jerant [45]	low		low		low		some concerns		high		some concerns	
Pratt [48]	low		some concerns		high		some concerns		some concerns		some concerns	
De Leo [51]	low		high		high		some concerns		some concerns		high	

Non-randomized articles	Confounding	Classification of interventions		Selection of participants		Deviations from intended interventions	Missing outcome data	Measurement of outcome		Selection of reported result		Overall
Stas [37]	serious	low		serious		low	serious	moderate		moderate		serious
Ogrodniczuk [38]	serious	low		serious		low	low	moderate		moderate		serious
Schlichthorst [40]	moderate	serious		serious		low	serious	moderate		moderate		serious
Daigle [42]	serious	serious		serious		low	moderate	moderate		moderate		serious
Nakao [46]	serious	low		serious		low	serious	moderate		moderate		serious
Calear [47]	serious	low		serious		low	serious	moderate		low		serious
Heisel [49]	serious	low		serious		moderate	moderate	moderate		serious		serious
Jackson [50]	low	low		serious		low	moderate	moderate		moderate		moderate

Across both randomized and non-randomized articles, measurement of outcome was commonly rated as some concerns or moderate risk of bias, respectively, as participant blinding was generally not feasible and outcomes were predominantly self-reported. In non-randomized articles, selection of participants into the study or analysis was frequently judged as serious risk of bias, reflecting the likelihood of selection mechanisms related to both intervention/exposure and outcome. Overall, the evidence base was characterized by substantial methodological limitations.

### Effectiveness of suicide prevention interventions targeting men

Effectiveness findings for suicide-related outcomes, depression, and help-seeking outcomes were synthesized across 17 articles. Table 3 provides an evidence summary by outcome, separating findings from RCTs and non-RCT designs. Detailed data extraction of outcome measures, assessment timepoints, and statistical results is provided in Supplementary Table S2.

**Table 3:** Effectiveness of suicide prevention interventions targeting men

Outcome	No. of studies (designs)	Key findings from RCTs (between-group effects)	Key findings from non-RCT designs	Summary of findings
Suicide deaths	*n* = 1 (1 RCT)	No events occurred	N/A	Insufficient
Suicide attempts	*n* = 3 (2 RCTs, 1 pre–post)	No significant effects	No significant changes	Limited
Suicidal ideation	*n* = 9 (5 RCTs, 1 NRCT, 1 cohort, 2 pre–post)	No significant effects	Significant reductions (*Hope service*, *MCMG*)	Study design-dependent
Additional suicide risk and suicidality outcomes	*n* = 3 (2 RCTs, 1 cohort)	No significant effects	Significant reductions (*EAP*)	Study design-dependent
Depression	*n* = 7 (4 RCTs, 2 cohorts, 1 pre–post)	No significant effects	Significant reductions (*Hope service*, *EAP*, *MCMG*)	Study design-dependent
Help-seeking behaviors	*n* = 5 (4 RCTs, 1 NRCT)	Significant effects (*MAPS*, *Man Therapy*)	No significant changes	Mixed
Help-seeking intentions	*n* = 8 (3 RCTs, 1 NRCT, 1 pre–post, 1 repeated cross-sectional, 2 cross-sectional)	Significant effects (*Man Up*)	Significant improvements (*Silence is Deadly*, *Get out of your head*)	Mixed
Help-seeking attitudes	*n* = 3 (1 RCT, 1 NRCT, 1 cross-sectional)	No significant effects	No significant changes	Limited
Help-seeking inhibition	*n* = 1 (1 RCT)	No significant effects	N/A	Insufficient

Non-RCT designs refer to study designs other than RCTs and included NRCTs, cohort, pre–post, repeated cross-sectional, and cross-sectional studies. Findings were summarized as “insufficient” when only one study assessed the outcome or no events occurred; “limited” when findings were consistently null across studies; “mixed” when results varied within RCTs; and “study design-dependent” when findings differed by study design (RCTs vs. non-randomized studies)

#### Suicide deaths

The outcome of suicide deaths was reported in only one included study, the RCT of *ICM* [51]. No suicide deaths occurred in either the intervention or control group during the 12-month follow-up period. Therefore, evidence was insufficient to determine intervention effectiveness on suicide mortality.

#### Suicide attempts

Three studies, including two RCTs and one pre–post evaluation, examined suicide attempts or closely related behaviors. The *CBSP* trial found no significant between-group effect on suicidal or self-injurious behaviors [48], and the *Contact+Connect* trial found no significant between-group effect on suicide attempts [43]. The pre–post evaluation of *SPW 2000* found no change in the proportion of participants reporting suicide attempts [42]. Overall, evidence regarding the effectiveness of interventions in reducing suicide attempts was limited.

#### Suicidal ideation

Suicidal ideation was the most frequently reported suicide-related outcome, assessed in nine studies, five of which were RCTs. Across these RCTs evaluating *Man Up*, *CBSP*, *Contact+Connect*, *Man Therapy*, and *ICM*, none demonstrated statistically significant between-group effects on suicidal ideation, regardless of risk of bias [35, 39, 43, 48, 51]. The controlled cohort study of *Hope service* did not demonstrate a significant between-group difference; however, a 55% reduction over time in the proportion of service users reporting suicidal ideation was observed in the overall sample [50]. The pre–post study of *MCMG* reported a statistically significant decrease in suicidal ideation following the intervention (*p* = 0.008, d = 0.56) [49]. The pre–post evaluation of *SPW 2000* found no change in the proportion of participants reporting suicidal ideation [42]. *Silence is Deadly* included suicidal ideation as a pre-specified outcome but did not report corresponding results [47]. Overall, evidence for intervention effectiveness in reducing suicidal ideation differed by study design, with RCTs consistently showing null between-group effects and some non-randomized studies reporting reductions over time.

#### Additional suicide risk and suicidality outcomes

Three studies, including two RCTs, reported additional suicide-related outcomes, including suicide probability, communication about suicide as a suicide risk indicator, and composite measures of suicidality. The *CBSP* trial found no significant between-group effect on suicide probability [48], and the *Contact+Connect* trial found no significant between-group effect on communication about suicide [43]. The controlled cohort study of *EAP* assessed suicidality and found no significant between-group difference; however, a statistically significant within-group reduction was observed in the intervention group (*p* = 0.039) but not in the control group [46].

Taking suicide-related outcomes together, no suicide deaths occurred in the only study reporting mortality. RCTs showed no significant between-group effects on suicide attempts, suicidal ideation, or other suicide-related outcomes, and some non-randomized studies reported reductions over time in certain outcomes.

#### Depression

Depression was assessed in seven studies, including four RCTs. Across these RCTs evaluating *Boys Do Cry*, *CBSP*, *Man Therapy*, and *ICM*, none demonstrated statistically significant between-group effects on depression, regardless of risk of bias [35, 41, 48, 51]. The two controlled cohort studies (*Hope service* and *EAP*) did not demonstrate significant between-group differences [46, 50]. Nevertheless, improvements over time were observed in three studies. The *Hope service* study reported a 49% reduction in depression scores in the overall sample [50], and the *EAP* study reported a statistically significant within-group reduction in the intervention group on overall depression severity (*p* = 0.001), including depressed mood (*p* = 0.004), which was not observed in the control group [46]. Furthermore, the pre–post study of *MCMG* reported a statistically significant decrease in depression following the intervention (*p* = 0.014, d = 0.57) [49]. Taken together, evidence for intervention effectiveness in reducing depression differed by study design, with RCTs consistently showing null between-group effects and all non-randomized studies that assessed this outcome reporting reductions over time.

#### Help-seeking behaviors

Five studies, including four RCTs and one NRCT, examined help-seeking behaviors, including disclosure of suicidal thoughts within healthcare encounters, engagement with formal (professional) and informal (non-professional) sources of support, and contact with health services.

The RCT evaluating *MAPS* reported a statistically significant between-group effect on discussion of suicidal thoughts during the subsequent primary care clinician visit (OR = 5.91, 95% CI [1.59, 21.94]) [45]. This effect was observed in a pilot sample (*n* = 62) and concerned a single secondary outcome, while the primary outcome and other pre-specified secondary outcomes were not reported. The *Man Therapy* RCT found no significant between-group effect on help-seeking behavior defined as beginning to see a counselor [35]. A secondary analysis of the same trial reported a statistically significant between-group effect on professional help-seeking behaviors (adjusted OR = 1.55, 95% CI [1.00, 2.40]), whereas no significant effect was observed for non-professional help-seeking behaviors [36]. These help-seeking outcomes were not pre-specified, and statistical significance was observed only after covariate adjustment using a *p* < 0.10 selection threshold.

The *Silence is Deadly* NRCT found no significant between-group difference in help-seeking behaviors for emotional problems from either formal or informal sources [47]. The RCT of *ICM* reported descriptive data indicating a higher proportion of participants in the intervention group reporting contact with health services following discharge [51]. *Boys Do Cry* included health service use as a pre-specified outcome but did not report corresponding results [41]. Overall, evidence for intervention effectiveness in improving help-seeking behaviors was mixed, with some RCTs reporting statistically significant between-group effects in the context of methodological and reporting concerns.

#### Help-seeking intentions

Help-seeking intentions were examined in eight studies, including three RCTs. They were commonly measured using the General Help-Seeking Questionnaire, which assesses the likelihood of seeking help from various sources under different problem contexts, including personal or emotional problems and suicidal ideation.

Two low-risk-of-bias RCTs assessed help-seeking intentions for personal or emotional problems but reported contrasting results: the *Boys Do Cry* RCT found no significant between-group effect [41], whereas the *Man Up* RCT demonstrated a statistically significant effect (coef. = 2.06, 95% CI [0.48, 3.63]) [39]. A high-risk-of-bias RCT evaluating *Man Therapy* found no significant between-group effect on help-seeking intentions for suicidal ideation [35].

The *Silence is Deadly* NRCT reported a statistically significant between-group difference in help-seeking intentions for emotional problems from a friend at follow-up (β = 0.493, *p* = 0.015), but not at post-intervention or from any other help source [47]. The pre–post study of *Get out of your head* found no significant change in overall help-seeking intentions or in intentions for personal or emotional problems; however, a statistically significant increase was observed for help-seeking intentions for suicidal ideation (*p* = 0.023, d = 0.15) [37]. The repeated cross-sectional study of the *Man Up* intervention found no significant difference in help-seeking intentions for personal or emotional problems between men surveyed before the campaign was aired and those surveyed after, regardless of exposure [40]. In the *SPW 2000* cross-sectional evaluation, no between-group differences were reported in help-seeking intentions if participants ever became suicidal or in help-seeking likelihood before attempting suicide [42]. In the *HeadsUpGuys* cross-sectional study, over half of respondents reported being more likely to seek professional help or informal support after visiting the website [38]. Overall, evidence for intervention effectiveness in improving help-seeking intentions was mixed, with findings varying by study design, problem context, help source, and assessment timepoint.

#### Help-seeking attitudes

Three studies, including one RCT, examined help-seeking attitudes. The *Man Therapy* RCT found no significant between-group effect on help-seeking attitudes toward professionals [35]. The *Silence is Deadly* NRCT found no significant between-group difference in help-seeking attitudes toward either professionals or trusted adults [47]. In the post-*SPW 2000* evaluation, no significant difference in help-seeking attitudes was observed between men exposed and not exposed to the campaign [42]. Overall, evidence regarding the effectiveness of interventions in improving help-seeking attitudes was limited.

#### Help-seeking inhibition

One study, the *Contact+Connect* trial, additionally assessed help-seeking inhibition, capturing feelings of shame, embarrassment, and perceived weakness related to seeking professional help for depression, and found no significant between-group effect [44].

Taken together, findings across help-seeking outcomes were mixed. Statistically significant improvements were observed for some aspects of help-seeking behaviors and intentions and were mostly reported in studies with substantial methodological limitations. No statistically significant improvements were observed for help-seeking attitudes or help-seeking inhibition.

### Gender-tailoring strategies in suicide prevention interventions targeting men

Among the 14 interventions reviewed, 11 incorporated gender-tailoring strategies addressing male-specific barriers to help-seeking and mental health. These barriers have been linked to masculine norms (e.g., stoicism and self-reliance) and associated stigma, which may discourage emotional expression and open communication. Identified gender-tailoring strategies were organized into seven categories with corresponding elements (Table 4). Detailed extracted gender-tailoring data are provided in Supplementary Table S5.

**Table 4:** Gender-tailoring strategies in suicide prevention interventions targeting men

Category	Elements	Interventions
Masculinity-informed intervention design and development	**Evidence-informed foundation**: drawing on theory and research on male help-seeking for psychological distress	*MAPS*
	**Expert-informed design**: incorporating input from experts in men’s health and men’s mental health	*Man Up*, *Get out of your head*
	**User co-production**: engaging men, particularly those with lived experience of depression and suicidality, in intervention design	*Man Therapy*, *Get out of your head*, *Contact+Connect*, *HeadsUpGuys*
	**Feedback-informed development**: incorporating feedback from men and relevant stakeholders in male suicide prevention, and responding to men’s changing needs	*Man Therapy*, *Contact+Connect*, *MAPS*
Masculinity-informed risk targeting	**Targeting male-salient life-context stressors associated with suicide risk**: addressing financial difficulties and retirement-related role loss	*Hope service*, *MCMG*
Masculinity-responsive content and messaging	**Challenging restrictive masculine norms and reducing stigma**: challenging stoicism and self-reliance norms that discourage emotional expression, open communication, and help-seeking; addressing stigma around men’s mental health problems, suicide, and help-seeking; and normalizing vulnerability and help-seeking as acceptable and appropriate for men	*Man Therapy*, *Get out of your head*, *HeadsUpGuys*, *Man Up*, *Boys Do Cry*, *SPW 2000*, *Contact+Connect*, *Silence is Deadly*
	**Enhancing mental health and suicide literacy**: improving recognition of distress and suicidality; understanding of risk and protective factors for depression and suicide; knowledge of self-care and recovery-oriented self-management; knowledge of professional help-seeking; and awareness of links between masculinity, help-seeking, mental health, and suicidality	*Get out of your head*, *Contact+Connect*, *Man Therapy*, *HeadsUpGuys*, *Boys Do Cry*, *Silence is Deadly*, *Man Up*
	**Promoting emotional expression and open communication**: encouraging expression of emotions and feelings; encouraging disclosure and discussion of personal issues, life’s challenges, mental health difficulties, and suicidal thoughts; and providing practical guidance on starting conversations about one’s own mental health problems and suicidality	*Boys Do Cry*, *Silence is Deadly*, *Man Therapy*, *MAPS*, *Get out of your head*
	**Promoting and reframing help-seeking**: encouraging informal and formal help-seeking; reframing help-seeking as a masculine strength; and positioning self-management on a pathway to peer and professional help-seeking	*Man Therapy*, *Get out of your head*, *HeadsUpGuys*, *Man Up*, *Boys Do Cry*, *SPW 2000*, *Contact+Connect*, *Silence is Deadly*
	**Promoting peer and mutual support**: encouraging male-to-male support, including both seeking and providing peer support; providing practical guidance on starting supportive conversations with peers experiencing mental health problems and suicidality; and encouraging sharing of intervention materials and resources with peers	*MCMG*, *Silence is Deadly*, *Man Up*, *HeadsUpGuys*, *Boys Do Cry*, *Get out of your head*
	**Strengthening social connectedness**,** hope**,** and resilience**: encouraging ongoing social connection with others and fostering hope, resilience, and recovery	*Contact+Connect*, *Man Therapy*, *HeadsUpGuys*, *MCMG*
Male-focused outreach and recruitment	**Setting-based recruitment**: recruiting men via male-oriented venues (e.g., sporting clubs, car shows), male-dominated disciplines (e.g., engineering, business), and male-focused events (e.g., a men’s retirement and leisure fair)	*Man Up*, *MCMG*, *Boys Do Cry*
	**Organization-based recruitment**: collaborating with a men’s suicide prevention organization and an expert advisory group specializing in men’s health	*Boys Do Cry*, *Man Up*
	**Peer-driven social diffusion**: encouraging informal sharing of intervention materials and participation experiences among peers, and snowball recruitment via existing participants	*Man Therapy*, *Boys Do Cry*
Male-congruent delivery modalities	**Group-based formats**: fostering cohesion, camaraderie, and peer support	*MCMG*
	**Self-guided web-based formats**: supporting anonymity, autonomy, and self-reliance	*HeadsUpGuys*, *Man Therapy*
Male representation and role modeling	**Male presence and visibility**: featuring exclusively male representation, including male facilitators and role models in intervention delivery and materials, ranging from everyday men to recognizable public figures	*Man Therapy*, *Get out of your head*, *HeadsUpGuys*, *Man Up*, *Boys Do Cry*, *Silence is Deadly*, *MCMG*
	**Behavioral role modeling**: demonstrating behaviors of expressing emotions, talking openly about problems, and seeking help	*Man Up*
	**Narrative role modeling**: sharing testimonials and success stories based on lived experiences of mental health challenges and suicidality, reflecting experiences of suicidal crisis, help-seeking, and positive life change	*Man Therapy*, *Get out of your head*, *HeadsUpGuys*, *Man Up*, *Boys Do Cry*, *Silence is Deadly*
Male-tailored communication and action orientation	**Male-tailored communication and framing**: using non-clinical intervention framing; male-oriented language (including colloquial vocabulary, humor, and metaphors); sports-based and occupation-specific framing; and adaptation to local community contexts	*MCMG*, *Silence is Deadly*, *Man Therapy*
	**Action-oriented and problem-solving approaches**: emphasizing action-oriented treatment styles and practical “how-to” strategies to support skills practice, problem-solving, and help-seeking	*Man Therapy*, *Get out of your head*, *Silence is Deadly*, *Boys Do Cry*

#### Masculinity-informed intervention design and development

Six interventions incorporated masculinity-informed considerations during intervention design and development. Intervention design drew on theory and research related to male help-seeking for psychological distress [45], incorporated input from experts in men’s health [39, 40] and men’s mental health [37], as well as direct input from men in the target population [35–37, 43, 44], particularly those with lived experience of depression and suicidality [38]. In addition, intervention development incorporated feedback from men [35, 36, 43, 44] and relevant stakeholders in male suicide prevention [45], with intervention resources continuing to expand in response to men’s changing needs [35, 36].

#### Masculinity-informed risk targeting

Two interventions targeted male-salient life-context stressors associated with suicide risk beyond psychological distress and suicidality. One intervention targeted men experiencing financial difficulties, including debt, employment, and welfare-related challenges, recognizing that economic recession and associated financial difficulties have been linked to increased suicide risk among men [50]. Another intervention targeted men concerned about or struggling with the transition to retirement, recognizing that this transition may reduce purposeful activity and a sense of routine, thereby increasing suicide risk among men who derive a sense of identity from work and career [49].

#### Masculinity-responsive content and messaging

Masculinity-responsive content and messaging were the most common gender-tailoring strategy, incorporated in ten interventions.

To deconstruct male-specific barriers, interventions explicitly challenged traditional masculine norms related to stoicism [39–41] and self-reliance [39, 40]. These included challenging expectations that men should avoid emotional expression [39, 40] (e.g., never crying [41] or expressing suffering [42]), keep difficulties to themselves [41], and cope alone by “toughing it out” [39, 40]. A recurring focus was challenging the notion that vulnerability [38] and help-seeking [39, 40] are incompatible with “being a man”. One campaign was named *Man Up* to challenge the conventional meaning of “man up” as “harden up” or “suck it up”, and used the tagline “Man Up, Speak Up” to raise awareness of the harm caused by men “toughing it out” [39, 40]. Intervention names such as *Boys Do Cry* and *Silence is Deadly* also served as subtle communicative cues.

Interventions also addressed stigma, myths, and stereotypes surrounding men’s mental health problems [35–38, 43, 44], suicide [35, 36], and help-seeking [38], including efforts to demystify how crisis support services operate [39, 40]. Normalization and social norming strategies were used to emphasize that mental health problems are common [47] and that help-seeking is acceptable and appropriate for men [38].

To build men’s capacity to recognize and respond to distress and suicidality, interventions aimed to enhance men’s mental health and suicide literacy through targeted information and practical “how-to” strategies [35–38]. This included improving recognition of signs of distress and suicidality [37] and providing information about risk and protective factors for depression and suicide [43, 44], for example through online self-assessments that helped men self-identify suicide risk and protective factors [35, 36]. Interventions also provided information on self-care [37] and recovery-oriented self-management for depression and suicidality [38], as well as information about professional help-seeking and access to relevant resources [37, 41, 43, 44]. Importantly, interventions also targeted literacy by highlighting how masculine norms can hinder early and effective help-seeking [47] and, in turn, worsen mental health and increase suicide risk [39, 40].

Interventions encouraged the expression of emotions and feelings [41] and promoted open communication, including disclosure and discussion of personal issues [47], life’s challenges [35, 36], mental health difficulties [41], and, in some cases, suicidal thoughts [45]. For example, the *Boys Do Cry* video ended with the call to action “When the going gets tough. Get Talking” [41]. Information was also provided on how to start conversations about one’s mental health problems and suicidality [37].

To enable action and strengthen protective resources, interventions placed an emphasis on promoting help-seeking [37, 41–44], aiming to empower men to seek help [35, 36]. Men were encouraged to seek informal and formal sources of support, such as peers [38, 47], professionals [38, 47], and other trusted adults [47]. Help-seeking was reframed as a sign of masculine strength and courage rather than weakness [35, 36, 38]. In one self-guided web-based intervention, self-management was positioned on a graduated pathway to peer and professional help-seeking, and both self-management and external help-seeking were framed as strengths [38]. Efforts to promote help-seeking integrated these elements, including norm-challenging and stigma-reduction messaging, validation of help-seeking, and the provision of information and support resources. In addition, interventions highlighted low help-seeking rates among men using statistics [47] and incorporated testimonials and success stories in which men shared positive help-seeking experiences [47] and described how reaching out for help had positively changed their life trajectory [39, 40].

Interventions also promoted peer and mutual support [49]. In the male-focused context of the interventions, references to supporting “others”, “someone”, or a “mate/friend” were understood as forms of peer support, reflecting support between men. Men were encouraged to seek support from peers [47], including speaking to them [47], opening up to them [39, 40], and reaching out for support [38]. At the same time, men were encouraged to provide support to peers [47], including encouraging peers to talk when experiencing mental health difficulties [41]. Men were also provided with strategies on how to take care of peers [37], how to start a conversation with a peer who is not doing well or expresses thoughts of suicide [47], as well as how to talk to a peer experiencing mental health difficulties or thinking about suicide [41]. Men were also encouraged to share intervention materials and resources with peers as a form of peer support [38].

In addition, interventions aimed to strengthen social connectedness by encouraging men to establish and maintain long-term contact with others [43, 44]. Interventions also sought to foster hope [35, 36, 38] and resilience [35, 36, 49], encouraging men to take action toward recovery [38].

#### Male-focused outreach and recruitment

Four interventions incorporated male-focused outreach and recruitment strategies. Recruitment targeted settings more likely to reach men, including male-oriented venues (e.g., sporting clubs [39] and car shows [49]), male-dominated university disciplines (e.g., engineering and business) [41], and purpose-designed male-focused events (e.g., a men’s retirement and leisure fair implemented within an intervention) [49]. Recruitment also involved collaboration with a men’s suicide prevention organization [41] and an expert advisory group specializing in men’s health, including representatives from men-focused community and service organizations and expert consultants [39]. In addition, peer-driven recruitment strategies were employed to promote participation, including encouraging men to share intervention materials and participation experiences with peers [35, 36], as well as snowball recruitment through existing male participants [41].

#### Male-congruent delivery modalities

Three interventions incorporated male-congruent delivery modalities. Group-based formats were used to foster cohesion and camaraderie, facilitating peer-based mutual support among participants [49]. Self-guided web-based formats were also used to ensure anonymity and privacy while aligning with men’s preferences for independence, autonomy, and self-reliance [38], allowing engagement without requiring immediate contact with mental health professionals [35, 36].

#### Male representation and role modeling

Seven interventions incorporated male representation and role modeling. Exclusively male figures were prominently featured as facilitators and role models in both intervention delivery and intervention materials (including, in one case, a fictional male host) [35–41, 47, 49]. These male figures ranged from everyday men from diverse backgrounds [39–41] to recognizable public figures, such as media personalities [39, 40] and athletes [47]. For example, one therapeutic intervention involved all-male participant groups facilitated by male-only facilitators [49], while a video-based intervention visually depicted a large and diverse group of men resembling a men’s support group [41].

Beyond representation alone, male role modeling was also incorporated. This included behavioral role modeling, such as men from all walks of life expressing emotions, talking openly about problems, and seeking help [39, 40], as well as narrative role modeling, whereby men shared personal stories, including testimonials and success stories. These narratives drew on men’s lived experiences of mental health challenges and suicidality [35–41, 47], highlighting experiences of suicidal crisis [39, 40], positive help-seeking experiences [47], and how reaching out for help had changed their life trajectory for the better [39, 40]. In one intervention, male role modeling was combined with social norming strategies [47].

#### Male-tailored communication and action orientation

Five interventions incorporated male-tailored communication and action orientation. Language and communication were adapted in male-tailored ways. This included framing interventions in non-clinical terms (e.g., advertising a “men’s group dealing with adjustment to retirement” rather than a “psychotherapy group”) [49] and using male-oriented language, including colloquial vocabulary (e.g., “mate” instead of “friend” in the Australian context) [47], as well as humor and metaphors familiar to men (e.g., car repair or fishing) [35, 36]. Communication materials, including those used during recruitment, further aligned with men’s identities through sports-based framing and occupation-specific framing for high-risk male occupational groups, as well as adaptation to local community contexts [35, 36].

In addition, action-oriented and problem-solving approaches were adopted. These included action-oriented treatment styles (focusing on skills practice, review, and feedback of progress to accomplish goals) [35, 36], as well as practical “how-to” strategies (e.g., how to recognize signs of suicidality [37], how to start a conversation about one’s mental health problems and suicidality [37], how to seek professional help and relevant resources [37], and how to help a peer [41, 47]).

## Discussion

To our knowledge, this systematic review is the first to synthesize the emerging evidence base on suicide prevention interventions targeting men. Overall, evidence for effectiveness was limited, with no clear effects on suicidality or depression and mixed findings for help-seeking. In addition, this review identified a wide range of gender-tailoring strategies incorporated in these interventions.

### Effectiveness of suicide prevention interventions targeting men

Findings from this review suggest that help-seeking was more responsive to intervention than depression or suicidality. Help-seeking is often considered a proximal and modifiable target that can be influenced through psychoeducation, social support, and barrier reduction [52, 53], and represents a critical first step in suicide prevention [54, 55]. In contrast, depression and suicidality are generally understood as more distal clinical outcomes influenced by multiple determinants [56, 57] and typically require sustained therapeutic effort to achieve meaningful impact [58]. Improvements in help-seeking alone may not necessarily translate into downstream effects on these clinical endpoints.

Findings further suggest that within the help-seeking domain, intentions and behaviors were more responsive to intervention than attitudes. Changes in intentions and behaviors may reflect relatively immediate responses to intervention content [59], whereas underlying help-seeking attitudes may be slower to change, particularly in the context of entrenched restrictive masculine norms and stigma surrounding help-seeking [13]. The pattern observed in this review does not fully align with the Theory of Planned Behavior, which conceptualizes attitudes as shaping intentions and subsequent behavior [31]. Observed improvements in intentions or behaviors may therefore be fragile and difficult to sustain over time in the absence of underlying attitudinal change [60].

Across the reviewed interventions, those that were primarily awareness-oriented, focusing on challenging restrictive masculine norms, reducing stigma, and improving mental health literacy, were more commonly associated with improvements in help-seeking (*Man Up*, *Silence is Deadly*, and *Get out of your head* [37, 39, 40, 47]). In contrast, interventions that were primarily therapeutically oriented, providing sustained professional support to strengthen coping and psychological adjustment, were more often associated with reductions in depression and suicidality (*Hope service*, *EAP*, and *MCMG* [46, 49, 50]). The *Man Therapy* website was primarily awareness-oriented and showed improvements in its secondary outcome of help-seeking, but not reductions in its primary outcomes of depression or suicidality [35, 36]. These patterns suggest that interventions tended to achieve the outcomes they were designed to influence. In particular, meaningful reductions in depression or suicidality may require a threshold of therapeutic intensity and sustained engagement [61, 62].

Many awareness-oriented interventions in this review were delivered through media-based formats. Website-based approaches, in particular, may not be best evaluated solely on their short-term impact on depression or suicidality. Instead, they may function as gateways to professional support or more comprehensive suicide prevention services, as suggested by the *Man Therapy* and *HeadsUpGuys* websites [35, 36, 38]. Notably, although media-based interventions showed potential to improve men’s help-seeking and offer advantages in scalability and resource efficiency, their effectiveness may have been constrained by limited exposure, superficial engagement, and insufficient reinforcement. Strengthening these factors may be necessary to initiate and sustain the attitude–intention–behavior pathway underlying help-seeking [31].

### Gender-tailoring strategies in suicide prevention interventions targeting men

This review identified a wide range of gender-tailoring strategies, consistent with existing literature [24, 27, 28]. These strategies form a multi-layered set of components that collectively address male-specific barriers to help-seeking and mental health, primarily related to restrictive masculine norms and stigma.

At the core, some strategies directly target these barriers through intervention content and messaging, including challenging restrictive masculine norms, reducing stigma, promoting emotional expression and open communication, and reframing help-seeking as a sign of strength, often delivered through male role modeling in behavioral and narrative forms. Additional content and messaging strategies, including enhancing mental health and suicide literacy, promoting peer and mutual support, and strengthening social connectedness, may address downstream consequences associated with masculine norms and stigma. Lower mental health literacy and social isolation, while not male-specific barriers per se, may be more pronounced among men [63]. Fostering hope and resilience may further support sustained engagement and recovery. At a broader level, masculinity-informed intervention design and development, together with risk targeting, shape how interventions are conceptualized and oriented. In addition, an “adhesive” layer of strategies may create enabling conditions that enhance relevance, acceptability, and engagement, through which restrictive masculine norms and stigma may be more readily addressed, including male-focused outreach and recruitment, male-congruent delivery modalities, male presence and visibility, and male-tailored communication and action orientation.

These identified strategies appear to engage with masculine norms in different ways. Some strategies, especially “adhesive” approaches, appear to work with dominant masculine norms. They may reduce identity threat, defensiveness, and resistance, and enhance the perceived safety of engagement, thereby lowering barriers to initial engagement [64]. However, if applied uncritically, such approaches may also risk reinforcing restrictive masculine norms [65]. In contrast, some strategies, especially those that directly target barriers within intervention content and messaging, appear to work against restrictive masculine norms. Through processes such as challenging, reframing, and normalizing, these strategies may facilitate deeper attitudinal and behavioral change. This tension can be understood through an alignment–transformation lens, which shares parallels with the distinction between gender accommodative and transformative approaches in the gender and development literature [66, 67], while focusing more specifically on how gender-tailoring strategies in suicide prevention interventions targeting men engage with masculine norms.

In practice, alignment- and transformation-oriented functions are often observed within a single strategy or across combinations of strategies. For example, exclusive male representation may enhance identification and perceived legitimacy, while behavioral and narrative role modeling may demonstrate that vulnerability and help-seeking are compatible with masculine identity. Peer and group-based approaches draw on masculine norms of camaraderie, while creating social contexts in which emotional expression, open communication, and help-seeking can be reinforced. Reframing help-seeking as masculine strength, courage, and as a rational step toward regaining self-control [17] draws on valued masculine ideals, while reshaping what it means to be a “real man”, such that strength may no longer equate with stoicism or rigid self-reliance. Together, these findings suggest that key gender-tailoring strategies may be combined in ways that reflect alignment–transformation processes, supporting both engagement and change in how masculinity relates to help-seeking and mental health.

Building on these findings, several gaps remain in gender-tailored suicide prevention strategies for men. First, most strategies focused on reducing barriers and facilitating initial help-seeking, with limited attention to sustaining change over time. Potential approaches to bridging entry and continuity may include expanding masculine identity repertoires to incorporate vulnerability and help-seeking as additional ways of being a man; integrating peer accompaniment or mentorship (e.g., “shadowing a man”) to support transitions from help-seeker to contributor and restore agency; and fostering hope and resilience by making recovery trajectories visible. Second, while sports- and hobby-based settings were used for recruitment, they were not leveraged as intervention environments. This represents a missed opportunity to embed psychological support within familiar and less stigmatizing social contexts, strengthen peer and social connections, and provide healthy emotional outlets and adaptive coping. Third, an intersectionality gap persists, as men were often treated as a homogeneous group. Masculinity was frequently approached as a shared set of norms and values, with limited attention to the plurality of masculinities shaped by intersecting social identities. In this sense, “gender-tailoring for men” has largely operated “across men” rather than being tailored to distinct groups of men. Finally, while interventions targeted financial distress and retirement transitions, broader structural barriers remained underexplored. When suicidality is driven by material insecurity (e.g., debt, unemployment, or housing instability), psychological support alone may be insufficient, underscoring the need for integrated multi-agency approaches combining socio-economic and psychological support [12].

### Intersection of intervention effectiveness and gender-tailoring

This review addressed intervention effectiveness and gender-tailoring in parallel. Effectiveness may reflect the value of gender-tailoring, and gender-tailoring may enhance relevance, acceptability, and engagement among men, which are often considered prerequisites for effectiveness [68]. Effectiveness alone does not explain why interventions work, and gender-tailoring alone does not demonstrate whether they work. Ideally, effectiveness should be examined in relation to the presence and degree of gender-tailoring. In line with theoretical assumptions, interventions targeting restrictive masculine norms and stigma may be more effective, and several interventions with positive effects included such components. However, examining whether and how gender-tailoring influences effectiveness remains constrained by the lack of established frameworks and measurement tools to define and assess gender-tailoring, including the extent to which masculine norms and stigma are addressed. As a result, the causal contribution of gender-tailoring to effectiveness remains to be tested.

### Limitations of the evidence base

Substantial heterogeneity across the included studies precluded meta-analysis and limited cross-study comparability. The overall robustness of the evidence was constrained by methodological limitations and a high risk of bias. Samples were largely self-selected, with participating men likely to have higher baseline help-seeking motivation and lower levels of depression or suicidality than the underlying target population of men. Such baseline differences were rarely adequately controlled for in non-randomized designs. Participation and completion rates were generally modest, reflecting persistent challenges in recruiting and retaining men in mental health and suicide prevention interventions, particularly in the context of restrictive masculine norms and stigma. Several studies also included relatively limited sample sizes, potentially constraining statistical power. Attrition may have been higher among disengaged or higher-risk participants. Furthermore, missing data were often inadequately handled. Participant blinding was generally not feasible, and outcomes were predominantly self-reported. In this context, restrictive masculine norms and stigma may have suppressed the reporting of vulnerability across suicidality, depression, and help-seeking, whereas intervention-related expectations may have inflated self-reported help-seeking behaviors and intentions. In addition, changes in rare suicide-related outcomes may have been difficult to detect due to insufficient sample sizes and follow-up periods. Depression outcomes may also have been inadequately captured, as generic depression scales were commonly used, and their items may not adequately reflect externalizing or masked depression presentations among men, which may be better captured by male-specific measures [19, 69].

Key gaps remain in the current evidence base. None of the included studies incorporated means-restriction components, representing a missed opportunity given men’s more frequent use of highly lethal methods [10, 11] and evidence supporting means-restriction strategies in suicide prevention [62]. Although the included interventions targeted several vulnerable groups of men, other important populations at elevated risk, including sexual minorities, ethnic minorities, and men living in rural areas, were underrepresented. In addition, although the review imposed no restrictions on context or setting, all included studies were conducted in high-income countries, limiting the generalizability of the findings to low- and middle-income countries, where gender gaps in suicide mortality are smaller but absolute male suicide rates remain high [2].

### Limitations of this review

Eligibility criteria may have narrowed the scope of included evidence. Mixed-gender studies were excluded, potentially omitting evidence on interventions that may be particularly relevant for men, including means-restriction strategies (e.g., restrictions on firearm access and bridge or railway barriers). Gatekeeper training interventions not directly targeting men were excluded despite their potential to reach men who may be reluctant to seek help through loved ones and other trusted social contacts. Studies relying on engagement-based outcome measures (e.g., website analytics) were excluded because the gender of respondents could not be reliably determined; however, such outcomes may still provide useful insights into help-seeking interest. In addition, qualitative studies, non-English articles, study protocols, conference abstracts, and grey literature were excluded, which may have further narrowed the breadth of perspectives captured.

Risk of bias was assessed at the study level rather than the outcome level. While outcome-level assessment may allow more granular judgments, a study-level approach was adopted for feasibility reasons and due to limited outcome-specific reporting in the included studies (e.g., outcome-specific missing data).

### Future directions

Future research should prioritize establishing a robust evidence base for suicide prevention interventions targeting men. Many male-targeted interventions already exist but remain unevaluated or insufficiently tested [25]. Future studies should therefore focus on rigorous effectiveness evaluations, including of existing initiatives, using methodologically robust study designs with low risk of bias, adequately powered samples, sufficient follow-up, and appropriate intervention duration and intensity. Establishing robust and credible evidence of effectiveness is a necessary precondition for any subsequent scale-up of male-specific suicide prevention interventions.

Advancing gender-tailored suicide prevention also requires greater conceptual precision. A critical next step is the development of a validated framework of gender-tailoring strategies. This process should begin with a synthesis of existing approaches, as undertaken in the present review, followed by expert consensus methods such as Delphi panels to delineate and prioritize core strategies. Subsequently, empirical designs such as factorial or SMART trials are needed to isolate and test the unique contribution of individual strategies. Together, conceptual consensus and empirical testing would enable the development of a practical, standardized checklist to guide intervention design, assess the quality of tailoring, and facilitate systematic comparison and benchmarking across interventions.

Many male suicide prevention interventions are grounded in the theoretical assumption that challenging restrictive masculine norms and stigma improves help-seeking, reduces depression, and ultimately lowers suicide risk [13, 14]. While parts of this epidemiological pathway are supported, the translation of these associations into intervention effects remains insufficiently tested. Future primary studies should move beyond parallel outcome reporting and explicitly examine mechanisms, for example through mediation analyses, to determine whether and how gender-tailoring strategies contribute to reductions in suicidality.

## Conclusions

This systematic review found limited evidence for the effectiveness of suicide prevention interventions targeting men, with no clear effects on suicidality or depression and mixed evidence regarding improvements in help-seeking. Interpretation of these findings was constrained by the heterogeneity of interventions and generally moderate to high risk of bias across studies. Nevertheless, the reviewed interventions incorporated a wide range of gender-tailoring strategies that collectively address male-specific barriers to help-seeking and mental health, particularly those related to restrictive masculine norms and stigma. Further development of gender-responsive, male-targeted approaches and more rigorous evaluation are needed to strengthen the evidence base. Given men’s disproportionately high suicide burden, advancing effective interventions remains an urgent public health priority aligned with the Sustainable Development Goals of reducing suicide mortality, addressing gender disparities, and promoting equity and reducing inequalities in mental health outcomes.

## Supplementary Information

Supplementary Material 1.

## Data Availability

The data that support the findings of this study are derived from publicly available studies included in this systematic review. Extracted data are presented in the main text and supplementary material.

## Abbreviations

CBSP: Cognitive Behavioural Suicide Prevention
EAP: Employee Assistance Programme
ICM: Intensive Case Management
MAPS: Men and Providers Preventing Suicide
MCMG: Meaning-Centered Men’s Groups
SPW 2000: Suicide Prevention Week 2000
PRISMA: Preferred Reporting Items for Systematic Reviews and Meta-Analyses
PROSPERO: International Prospective Register of Systematic Reviews
RCT: Randomized controlled trial
NRCT: Non-randomized controlled trial
RoB 2: Revised Cochrane risk-of-bias tool for randomized trials
ROBINS-I V2: Risk Of Bias In Non-randomized Studies – of Interventions, Version 2
