# Supplementary material for: Effectiveness and gender-tailoring of suicide prevention interventions for men: a systematic review
Source: BMC Public Health. 2026 Jun 23;26:2129. doi: 10.1186/s12889-026-28269-1 (PMC13359848; doi:10.1186/s12889-026-28269-1)
Supplement: Supplementary file 1 — Supplementary Material 1. [file 12889_2026_28269_MOESM1_ESM.pdf]

Supplementary Table S1. Studies excluded after full-text screening with reasons for exclusion

No.	Title of article	Reason(s) for exclusion
	**Interventions not targeting suicidality**	
1	*A Preliminary Examination of the "Real Men. Real Depression" Campaign*	Intervention does not aim to reduce or prevent suicidality; it is a depression awareness campaign.
2	*Addressing Suicidal Ideations Through the Realization of Meaningful Personal Goals*	1) Intervention does not aim to reduce or prevent suicidality; it is a personal goal intervention.<br>2) Mixed-gender study
3	*An Application of Social Marketing Theory to Develop a Social Marketing Campaign to Address Mental Health Literacy and Help-Seeking Behavior Among Male College Students*	1) Intervention does not aim to reduce or prevent suicidality; it is a social marketing campaign to improve mental health literacy and help-seeking behavior.<br>2) Undesired qualitative study design with 3) Irrelevant outcome measures
4	*An Examination of a Modified START NOW Dialectical Behavior Therapy-Based Intervention and a Behavioral Level System on Male Inmate Misbehavior, Aggressive Behavior, and Suicide Precaution Status*	Intervention does not aim to reduce or prevent suicidality; it is a behavioral intervention targeting inmate disruptive, violent, and aggressive behavior.
5	*Changes in Depression, Cognitive Distortions, and Self-Esteem as a Function of Change in Mindfulness Among Adult Male Inmates*	Intervention does not aim to reduce or prevent suicidality; it is a mindfulness intervention.
6	*Effectiveness of a Brief Stress Management Intervention in Male College Students*	Intervention does not aim to reduce or prevent suicidality; it is a brief stress management intervention.
7	*Exploring the Effectiveness of an Integrated Exercise/CBT Intervention for Young Men's Mental Health*	Intervention does not aim to reduce or prevent suicidality; it is an integrated exercise/ cognitive behavioral therapy (CBT) intervention for general mental health.
8	*Improving Mental Health Help-Seeking Among Male University Students: A Series of Gender-Sensitive Mental Health Feasibility Interventions*	Intervention does not aim to reduce or prevent suicidality; it includes three gender-sensitive mental health interventions to promote help-seeking.
9	*Pilot Evaluation of the Coping Course: A Cognitive-Behavioral Intervention to Enhance Coping Skills in Incarcerated Youth*	Intervention does not aim to reduce or prevent suicidality; it is a cognitive-behavioral intervention to improve coping skills.
10	*The Effect of Structured Education on Self-Esteem and the Suicide Probability of Male Adolescents Living in Orphanages*	Intervention does not aim to reduce or prevent suicidality; it is an educational program to improve self-esteem and hope.
11	*The Effectiveness of Acceptance and Commitment Therapy on the Meaning of Life and Psychological Capital of Male Students with Suicidal Ideation*	1) Intervention does not aim to reduce or prevent suicidality; it is an acceptance and commitment therapy (ACT).<br>2) No desired outcomes: it measures meaning of life and psychological capital.
12	*The Impact of a Depression Awareness Campaign on Mental Health Literacy and Mental Morbidity Among Gay Men*	Intervention does not aim to reduce or prevent suicidality; it is a depression awareness campaign.
13	*The Ripple Effect: A Digital Intervention to Reduce Suicide Stigma Among Farming Men*	1) Intervention does not aim to reduce or prevent suicidality; it is a suicide stigma reduction intervention.<br>2) No desired outcomes: it measures suicide stigma and suicide literacy.
14	*The Role of First-Person Depression Storytelling Online Video on Men's Self-Stigma of Seeking Help, Traditional Masculinity Ideology, and Psychological Help-Seeking Attitudes*	1) Intervention does not aim to reduce or prevent suicidality; it is aimed at depression.<br>2) Non-intervention effectiveness study; it is a mediation study testing how traditional masculinity ideology affects help-seeking attitudes and whether self-stigma mediates that relationship across three different conditions.
	**Indirect / gatekeeper interventions**	
15	*Comparison of the Effects of Four Suicide Prevention Programs for Family and Friends of High-Risk Suicidal Men Who Do Not Seek Help Themselves*	Indirect (gatekeeper) intervention: The intervention targets gatekeepers, and outcomes are collected via the gatekeepers regarding suicidal men.
	**Population / sample issues**	
16	*A Longitudinal Assessment of Two Suicide Prevention Training Programs for the Construction Industry*	1) Mixed-gender study<br>2) GAT and MAT are gatekeeper training programs, not direct suicide prevention interventions
17	*Cognitive Therapy for the Prevention of Suicide Attempts: A Randomized Controlled Trial*	Mixed-gender study
18	*Effects of Suicide Prevention Videos Developed by and Targeting Adolescents: A Randomized Controlled Trial*	Mixed-gender study

19	*Evaluating the SOS Suicide Prevention Program: A Replication and Extension*	Mixed-gender study
20	*Evaluating the Effectiveness of a Website About Masculinity and Suicide to Prompt Help-Seeking*	1) Unclear participant information: It is not possible to distinguish whether study respondents were men or women.<br>2) Google Analytics data (e.g., outbound clicks and downloads) are a distant and indirect proxy for help-seeking and do not adequately capture changes in psychological state (e.g., depressive symptoms or suicidal ideation), clinical suicide risk (e.g., suicide attempts), or help-seeking attitudes, intentions, and behaviors.
21	*Evaluating the Effectiveness in Initiating Help-Seeking Behaviors by Exposure to an Adult Male Public Service Announcement*	1) Unclear participant information: It is not possible to determine whether the intervention was sent to men or whether men responded to the study.<br>2) Google Analytics data (e.g., click-through rate) are a distant and indirect proxy for help-seeking and do not adequately capture changes in psychological state (e.g., depressive symptoms or suicidal ideation), clinical suicide risk (e.g., suicide attempts), or help-seeking attitudes, intentions, and behaviors.
	**Study design / outcome issues**	
22	*Can Watching a Television Documentary Change the Way Men View Masculinity?*	1) Undesired qualitative study design with 2) Irrelevant outcome measures
23	*Enhancing Psychological Resiliency in Older Men Facing Retirement with Meaning-Centered Men's Groups*	No results reported
24	*Meaning-Centered Men's Groups (MCMG) for the Transition to Retirement*	No desired outcomes
	**Publication / accessibility issues**	
25	*Effectiveness of Acceptance and Commitment Therapy (ACT) on Levels of Self-Criticism and Suicidal Thoughts in Adolescents with Self-Harm*	Full text not available in English
26	*Effectiveness of Unity-Oriented Psychology Approach on Depression and Suicidal Tendencies in Male Students*	Full text not available in English
27	*HeadsUpGuys: Canadian Online Resource for Men with Depression*	Undesired publication type: it is a commentary.
28	*Correction to "Effectiveness of Man Therapy to Reduce Suicidal Ideation"*	Correction or erratum

Supplementary Table S2. Detailed data extraction of study characteristics and intervention effectiveness

First author	Participant characteristics (age range)	Intervention characteristics	Intervention/exposure group or condition	Comparison group or condition	Effectiveness outcomes (measures & timepoints)	Key results	Funding sources
Frey	working-aged men (25–64) in Michigan, screened positive for suicide risk or were at moderate to high depression risk	*Man Therapy*:<br>a website that frames its approach around hope and resilience, integrating humor within a positive psychology/positive masculinity framework and using a fictional therapist persona, Dr. Rich Mahogany. It provides online self-assessments to identify suicide risk and protective factors and encourage taking appropriate responses for help-seeking, as well as "manly mental health information, facts and tips", "man therapies", and videos of men with lived experience.<br>*Man Therapy* aims to help men explore how gender socialization influences relationships and mental health, explore gender-specific issues linked to risk for suicide and mental health problems (especially depression), change cultural norms to reduce stigma about mental health and suicide, empower men to seek help by reframing help-seeking as a sign of strength rather than weakness, and reduce depression and suicide risk.<br>*duration: 3 months	*Healthy Men Michigan + Man Therapy*	*Healthy Men Michigan* only<br>a website for depression and suicidal ideation screening, with immediate risk feedback and referral information	1.suicidal ideation: C-SSRS Screen Version<br>2.depression: HANDS<br>3.help-seeking behavior: reporting began seeing a counselor (yes/no)<br>4.help-seeking intentions for suicidal ideation: GHSQ<br>5.help-seeking attitudes toward professionals: ATSPPH-SF<br>*assessment: baseline, 2 weeks (expanded survey), 12 weeks (post-intervention)	**suicidal ideation**<br>• non-sig. between-group effect (slope difference: b = −0.05, p = 0.32, 95% CI [−0.24, 0.06])<br>**depression**<br>• non-sig. between-group effect (slope difference: b = −0.03, p = 0.16, 95% CI [−0.08, 0.01])<br>**help-seeking behavior – begin seeing a counselor**<br>• non-sig. between-group effect: at baseline, 9% of participants in each group reported they had begun seeing a counselor; post-intervention, 17% in the intervention group vs. 19% in the control group<br>**help-seeking intentions for suicidal ideation**<br>• non-sig. between-group effect (data NR)<br>• sig. within-group increase in intervention group (MD = 2.29, SD = 9.97, p < 0.001, 95% CI [0.75, 3.83]); control group data NR<br>**help-seeking attitudes toward professionals**<br>• non-sig. between-group effect (data NR)<br>• sig. within-group increase in intervention group (MD = 3.77, SD = 6.92, p = 0.004, 95% CI [2.70, 4.84]); control group data NR	CDC's National Center for Injury Prevention and Control
Gilgoff					1.professional help-seeking behaviors: 8-item checklist, e.g., locate professionals, make appointments, assessed by professionals, therapy/ counseling, professional-led support groups, other<br>2.non-professional help-seeking behaviors: 5-item checklist, e.g., peer groups, online forums/ chat rooms, trusted internet information, friends/family, other<br>*assessment: 2 weeks (expanded survey after baseline), 12 weeks (post-intervention)	**help-seeking behaviors – professional**<br>• sig. between-group effect in adj. model (OR = 1.55, p = 0.049, 95% CI [1.00, 2.40]); non-sig. in unadj. model (OR = 1.43, p = 0.096, 95% CI [0.94, 2.18])<br>**help-seeking behaviors – non-professional**<br>• non-sig. between-group effect in adj. model (OR = 1.11, p = 0.658, 95% CI [0.69, 1.80]) and unadj. model (OR = 1.18, p = 0.497, 95% CI [0.74, 1.88])	
Stas	men (19–72) from Flanders	*Get out of your head / Kom uit je kop*:<br>a campaign that begins with two campaign videos directing viewers to a dedicated website. The website features four video testimonials from men who have struggled with mental health issues, and provides information on recognizing signs of suicidality, self-care and caring for others, starting conversations about mental health problems and suicidality, and seeking professional help and other resources. It aims to improve men's mental health and reduce stigma, improve recognition of warning signs, and increase help-seeking and peer support.<br>*duration: 3 days	*Get out of your head*	no control group	1.help-seeking intentions: GHSQ<br>*assessment: baseline, 3 days (post-intervention)	**help-seeking intentions**<br>• non-sig. pre–post (within-group) change (MD = 1.48, 95% CI [−0.11, 3.08], p = 0.068, d = 0.11), from M = 64.33, SE = 1.01 to M = 65.81, SE = 1.13<br>among two subscales<br>• non-sig. pre–post (within-group) change in help-seeking intentions for personal/ emotional problems (MD = 0.32, 95% CI [−0.55, 1.19], p = 0.471, d = 0.05), from M = 33.66, SE = 0.48 to M = 33.98, SE = 0.55<br>• sig. pre–post (within-group) increase in help-seeking intentions for suicidal ideation (MD = 1.21, 95% CI [0.17, 2.25], p = 0.023, d = 0.15), from M = 30.67, SE = 0.60 to M = 31.88, SE = 0.68	Flemish Government – Department of Welfare, Public Health and Family
Ogrodniczuk	men (18–79) recruited after visiting the intervention website	*HeadsUpGuys*:<br>a website providing targeted information, practical advice, and strategies for managing and recovering from depression and suicidality, promoting a graduated approach from self-management to peer/professional help-seeking, and a strength-based approach that frames both as masculine strength<br>*duration: n/a	*HeadsupGuys*	no control group	1.help-seeking intentions: reporting whether they were more likely to seek professional/informal support after intervention (yes/no/already sought help)<br>*assessment: after visiting the website	**help-seeking intentions**<br>• among men who had not previously sought mental health professional support (n = 321), 65.7% (n = 211) reported that they were more likely to seek professional support after visiting the website, while 34.3% (n = 110) reported being not more likely<br>• among men who had not previously sought help from a friend or family member (n = 334), 55.4% (n = 185) reported that they were more likely to seek informal support after visiting the website, while 44.6% (n = 149) reported being not more likely	Canada Research Chair Program (support to J.L. Oliffe)
King	men (18+) not at active suicide risk	*Man Up*:<br>a three-part documentary (3×1 hour) hosted by Gus Worland, exploring the link between masculinity, help-seeking, men's mental health, and suicidality. Episode 1 covers men's experiences with suicidal crises, what led them to this point, and what got them through; Episode 2 highlights efforts by organizations and individuals encouraging men to open up to their mates to combat suicide; Episode 3 focuses on the creation	*Man Up*	*Test Your Brain*<br>a three-part documentary (3×1 hour) focusing on the inner workings of the brain, especially attention, sensory perception, and memory, through interactive experiments	1.suicidal ideation: ASIQ<br>2.help-seeking intentions for personal or emotional problems: GHSQ<br>*assessment: baseline, 4 weeks after viewing the documentary (follow-up)	**suicidal ideation**<br>• non-sig. between-group effect (coef. = 1.03, 95% CI [−1.39, 3.44], p = 0.405, SMD = 0.04)<br>• within-group: intervention group M = 12.75, SD = 13.11 → M = 13.46, SD = 13.93 (MD = 0.74); control group M = 12.40, SD = 17.26 → M = 12.20, SD = 16.39 (MD = −0.20)<br>**help-seeking intentions for personal or emotional problems**<br>• sig. between-group effect (coef. = 2.06, 95% CI [0.48, 3.63], p = 0.011, SMD = 0.13)<br>• within-group: intervention group M = 44.64, SD = 10.03 → M = 47.53, SD = 10.80 (MD = 2.89); control group M = 43.89, SD = 9.37 → M = 44.96, SD = 8.90 (MD = 1.07)	Movember Foundation

Schlichthorst	males of any age (69% between 25 and 54)	of a campaign ad with the tagline "Man Up, Speak Up" to raise awareness about the damage caused by men "toughing it out".<br>*duration: 3 hours	completed survey after *Man Up* aired and viewed it	completed survey before *Man Up* aired<br><br>completed survey after *Man Up* aired but had not viewed it	1.help-seeking intention for personal or emotional problems: adapted GHSQ (simplified overall item assessing general help-seeking likelihood, rather than source-specific subitems)<br>*assessment: before and after *Man Up* aired	**help-seeking intention for personal or emotional problems**<br>• non-sig. difference between men who completed survey after *Man Up* aired and viewed it (M = 4.18, SD = 1.77) and those who completed survey before *Man Up* aired (M = 4.16, SD = 1.79) (adj. coef. = 0.09, 95% CI [−0.17, 0.35], p = 0.485; unadj. coef. = 0.02, 95% CI [−0.21, 0.25], p = 0.853)<br>• non-sig. difference between men who completed survey after *Man Up* aired but had not viewed it (M = 3.85, SD = 1.76) and those who completed survey before *Man Up* aired (adj. coef. = −0.22, 95% CI [−0.57, 0.12], p = 0.209; unadj. coef. = −0.31, 95% CI [−0.62, 0.00], p = 0.052)	Movember Foundation
Nicholas	men (18–87)	*Boys Do Cry*:<br>a 4-minute video adaptation of a well-known song, with lyrics emphasizing how men are typically discouraged from discussing their difficulties and encouraging men to express their feelings and seek support from others when experiencing mental health difficulties. The video ends with a call to action, "When the going gets tough. Get Talking", and a link to a website with mental health resources (not active during the trial).<br>*duration: 1 week (the 4-minute video can be re-watched)	*Boys Do Cry*	*Redesign My Brain*<br>a 4-minute video focusing on brain health, in which a central figure is taught table tennis by two champions, aiming to improve cognitive processing speed	1.depression: MDRS-7<br>2.help-seeking behavior – health service use: HSUQ<br>3.help-seeking intentions for personal or emotional problems: GHSQ<br>*assessment: baseline, 1 week post-baseline (post-intervention), and 4 weeks after the 1-week assessment (follow-up)	**depression**<br>• non-sig. between-group effect at follow-up (modeled MD = −0.21, 95% CI [−0.64, 0.22], p = 0.335, modeled SMD = −0.09)<br>• within-group: intervention group M = 11.26, SD = 2.36 → M = 10.61, SD = 2.29; control group M = 10.98, SD = 2.39 → M = 10.83, SD = 2.37<br>**help-seeking behavior – health service use** not reported<br>**help-seeking intentions for personal or emotional problems**<br>• non-sig. between-group effect at post-intervention (modeled MD = 0.62, 95% CI [−1.11, 2.35], p = 0.485, modeled SMD = 0.06) and at follow-up (modeled MD = 1.32, 95% CI [−0.39, 3.04], p = 0.130, modeled SMD = 0.14)<br>• within-group: intervention group M = 45.28, SD = 10.11 → M = 47.33, SD = 9.60 → M = 47.66, SD = 9.60; control group M = 45.70, SD = 9.47 → M = 46.59, SD = 9.48 → M = 46.29, SD = 9.04	Medical Research Future Fund; Suicide Prevention Australia
Daigle	a representative sample of men (18+) in Quebec	*Suicide Prevention Week / SPW*:<br>an annual public health (media) campaign targeting men aged 20–40, aiming to change the behaviors of suicidal individuals and the public will through media outreach and community-based activities (e.g., local meetings and conferences). *SPW 2000* promoted men's right to feel pain and seek help. The slogan, "Pain is not gender-specific – yet 80% of suicides are committed by men", aimed to change the belief that men should not feel pain or express suffering.<br>*duration: 1 week	exposed to *SPW 2000*	non-exposed to *SPW 2000*	1.suicide attempts: whether had attempted suicide in the past 2 weeks<br>2.suicidal ideation: whether had thought about committing suicide in the past 2 weeks<br>*assessment: before and after *SPW 2000*<br>3.help-seeking intention if ever became suicidal: inspired by Ajzen & Fishbein (1980)<br>4.help-seeking likelihood before committing suicide: rated on a 0–10 Likert scale<br>5.help-seeking attitudes: 6 items, inspired by Dulac (1997) and Canetto & Lester (1995) on masculinity<br>*assessment: after *SPW 2000*	**suicide attempts**<br>• no change: 0.2% of participants reported suicide attempts at both pre- and post-*SPW 2000*<br>**suicidal ideation**<br>• no change: 0.6% of participants reported suicidal ideation at both pre- and post-*SPW 2000*<br>**help-seeking intentions**<br>• non-sig. between-group difference in help-seeking intention if participants ever became suicidal (67.0% in exposed group vs. 63.7% in non-exposed group reported help-seeking intention, p NR)<br>• no between-group difference in help-seeking likelihood before committing suicide (data NR) (Note: interpreted as before "attempting" suicide)<br>**help-seeking attitudes**<br>• non-sig. between-group difference (exposed group: M = 7.23, SD = 1.58; non-exposed group: M = 7.06, SD = 1.61; t = 1.28, p NR)	Québec Ministry of Health and Social Services
Milner (2019)	male construction workers (18+) in Victoria, registered with Incolink	*Contact+Connect*:<br>a brief contact intervention delivering six weekly rich text messages via the Whispir™ system to smartphones. Messages link to microsites, videos, and digital wallet cards. Content includes information on stigma, mental health, help-seeking resources, and encouragement to establish and maintain long-term contact with others.<br>*duration: 6 weeks	*Contact+Connect*	waitlist control	1.suicide attempts: adapted SBQ-R<br>2.suicidal ideation: adapted SBQ-R<br>3.communication about suicide: adapted SBQ-R<br>*assessment: baseline, 6 weeks (post-intervention)<br><br>**follow-up analysis at 12 weeks canceled due to limited responses	**suicide attempts**<br>• non-sig. between-group effect (IPTW MD = 0.08, 95% CI [−0.10, 0.27], p = 0.377)<br>• non-sig. within-group change in both groups: intervention group MD = 0.08, 95% CI [−0.05, 0.22]; control group MD = −0.01, 95% CI [−0.12, 0.12]<br>**suicidal ideation**<br>• non-sig. between-group effect (IPTW MD = 0.19, 95% CI [−0.03, 0.41], p = 0.095)<br>• within-group: sig. decrease in control group (MD = −0.29, 95% CI [−0.44, −0.14]) vs. non-sig. change in intervention group (MD = −0.10, 95% CI [−0.27, 0.07])<br>**communication about suicide**<br>• non-sig. between-group effect (IPTW MD = 0.19, 95% CI [−0.01, 0.39], p = 0.064)<br>• non-sig. within-group change in both groups: intervention group MD = 0.07, 95% CI [−0.07, 0.23]; control group MD = −0.11, 95% CI [−0.25, 0.02]<br>*Above data from IPTW analyses. Similar results observed across three analytic approaches (unadj., adj., and IPTW).	Beyond Blue – Stride Program
Milner (2017)					1.help-seeking inhibition: SSDS<br>*assessment: same as above	**help-seeking inhibition**<br>• non-sig. between-group effect (adj. MD = −0.03, 95% CI [−0.74, 0.68], p = 0.931; unadj. MD = 0.03, 95% CI [−0.66, 0.73], p = 0.917)<br>• non-sig. within-group change in both groups: intervention group (adj. MD = −0.37, 95% CI [−0.90, 0.16]; unadj. MD = −0.31, 95% CI [−0.82, 0.20]) and control group (adj. MD = −0.34, 95% CI [−0.86, 0.18]; unadj. MD = −0.35, 95% CI [−0.85, 0.15])	

Jerant	middle-aged men (35–74) with recent (4 weeks) active suicidal thoughts, visiting a primary care clinician (PCC) at a single California health system	*Men and Providers Preventing Suicide / MAPS*:<br>a tailored interactive computer program completed in-office before a PCC visit to encourage discussion of recent suicidal thoughts. It allows anonymous disclosure initially and provides empathetic texts and videos acknowledging suicidal thoughts. Three motivational modules guide users in preparing for disclosure using video clips modeling discussion strategies, collaborative care planning, and ongoing monitoring with adjustment of care plans. Content is tailored to user responses, knowledge, perceptions, and suicide risk factors, with optional material for further exploration.<br>*duration: 15–20 minutes	*MAPS*	5 minutes of content including a 3-minute sleep hygiene informational video, a non-tailored text screen encouraging discussion of suicidal thoughts with PCC, and general information about suicide risk factors and support resources	1.help-seeking behavior – discussion of suicidal thoughts during PCC visit (yes/no)<br>*assessment: after intervention and PCC visit	**help-seeking behavior – discussion of suicidal thoughts during PCC visit**<br>• sig. between-group effect (OR = 5.91, 95% CI [1.59, 21.94], p = 0.008; nesting-adjusted predicted effect: 71% for *MAPS* vs. 30% for control)	CDC's National Center for Injury Prevention and Control; UC Davis, Behavioral Health Center of Excellence (supporting A. Jerant); UC Davis, Department of Family and Community Medicine (supporting A. Jerant)
Nakao	intervention group: male employees (22–38) from an information service company in Tokyo<br><br>control group: men from an affiliated company at the same worksite	*Employee Assistance Programme / EAP*:<br>a workplace initiative offering free, anonymous counseling with psychologists via email or phone; referrals to an affiliated psychiatric clinic; and five annual job-related mental health seminars covering self-care, effect of excessive work on health, early detection of distressed colleagues, occupational maladjustment, and communication skills<br>*duration: 2 years	periodic health examinations + *EAP*	periodic health examinations only	1.suicidality: HAM-D ("Suicide" item)<br>2.depression: HAM-D (total score, including a "Depressed mood" item)<br>*assessment: baseline, 2 years (post-intervention)	**suicidality (HAM-D "suicide" item)**<br>• non-sig. between-group difference (data NR)<br>• sig. within-group decrease in intervention group (p = 0.039); non-sig. change in control group (p > 0.05)<br>**depression (HAM-D total score)**<br>• non-sig. between-group difference (data NR)<br>• sig. within-group decrease in intervention group (median = 6, range 0–27 → median = 5, range 0–23, p = 0.001); non-sig. change in control group (median = 5, range 1–13 → median = 5, range 1–10, p = 0.173)<br>depressed mood (HAM-D item)<br>• non-sig. between-group difference (data NR)<br>• sig. within-group decrease in intervention group (p = 0.004); non-sig. change in control group (p > 0.05)	The Occupational Health Promotion Foundation; Ministry of Health, Labour and Welfare of Japan – Health and Labour Sciences Research Grants
Calear	male adolescents (16–18+) in year 11 and 12 from government colleges and private secondary schools in the Australian Capital Territory	*Silence is Deadly*:<br>a school-based program that promotes early help-seeking for emotional problems. The core component is a 45–60-minute psychoeducational presentation covering statistics on mental health, suicide, and help-seeking, and how masculine norms can hinder early and effective help-seeking. The program uses male role modeling and social norming, delivered by trained Menslink presenters and reinforced by local celebrity athletes who share positive help-seeking experiences, challenge traditional masculine norms, and promote open communication. It emphasizes both seeking and providing peer support ("help a mate" strategies) and encourages seeking help from professionals and other trusted adults. The program is supported by a website, videos, and informational handouts.<br>*duration: 45–60 minutes	*Silence is Deadly*	waitlist control	1.suicidal ideation: YRBS<br>2.help-seeking behaviors for emotional problems: AHSQ<br>3.help-seeking intentions for emotional problems: GHSQ<br>4.help-seeking attitudes toward professionals: ATSPPH-SF<br>5.help-seeking attitudes toward trusted adults: drawn from the Sources of Strength trial (Wyman et al., 2008)<br>*assessment: baseline, 1–2 weeks (post-intervention), 6–12 weeks after presentation (follow-up)<br><br>**Help-seeking intentions and behaviors for personal problems were omitted from the analysis	**suicidal ideation** not reported<br>**help-seeking behaviors for emotional problems**<br>• from formal sources: no robust between-group difference; a nominally sig. between-group difference at follow-up in the primary analysis (β = 0.196, t = 2.034, p = 0.042), but became non-sig. after sensitivity analysis adjusting for demographic variables<br>• from informal sources: non-sig. between-group difference<br>**help-seeking intentions for emotional problems**<br>• from a friend: sig. condition × time interaction at follow-up (β = 0.493, t = 2.437, p = 0.015), which remained sig. after sensitivity analysis; non-sig. between-group difference at post-intervention<br>• from other sources of help: non-sig. between-group difference at post-intervention and follow-up<br>**help-seeking attitudes**<br>• toward professionals: non-sig. between-group difference<br>• toward trusted adults: non-sig. between-group difference	Australian Rotary Health; National Health and Medical Research Council (supporting A.L. Calear and P.J. Batterham); Australian Research Council (supporting M. Banfield)
Pratt	male prisoners (21–60) from a single site in Northwest England, identified as at risk of suicidal behavior within the past month under the HM Prison Service's Assessment, Care in Custody and Teamwork (ACCT) system	*Cognitive Behavioural Suicide Prevention / CBSP*:<br>a group therapy modularized into five components: attention broadening, cognitive restructuring, mood management and behavioral activation, problem-solving training, and improving self-esteem and positive schema. It includes up to 20 sessions (up to 1 hour each), initially delivered twice weekly and then reducing to once weekly. Sessions are provided by two trial therapists (clinical psychologists experienced in CBT) who receive initial *CBSP* training and ongoing case supervision.<br>*duration: 4 months	TAU + *CBSP*	TAU only<br>usual prison care/support under the ACCT system, including initial risk assessment, risk management plan, fortnightly reviews, Mental Health In-Reach team referral, and medication, but without psychological therapy	1.suicidal or self-injurious behavior: number of episodes in the past six months based on prison records<br>2.suicidal ideation: BSSI<br>3.suicide probability: SPS<br>4.depression: BDI-II<br>*assessment: baseline, 4 months (post-intervention), 6 months (follow-up)	**suicidal or self-injurious behavior**<br>• non-sig. between-group effect at 6 months (treatment effect = −0.72, SE = 0.47, 95% CI [−1.71, 0.09], p = 0.162)<br>• within-group: intervention group M = 1.06, SD = 2.10 → M = 0.58, SD = 1.52 (6 months); control group M = 1.39, SD = 3.28 → M = 1.48, SD = 3.23<br>**suicidal ideation**<br>• non-sig. between-group effect at 6 months (data NR)<br>• within-group: intervention group M = 13.2, SD = 10.8 → M = 5.8, SD = 9.9 (4 months) → M = 6.6, SD = 10.4 (6 months); control group M = 14.5, SD = 11.2 → M = 6.7, SD = 10.5 → M = 7.7, SD = 11.4<br>**suicide probability**<br>• non-sig. between-group effect at 6 months (data NR)<br>• within-group: intervention group M = 86.9, SD = 19.9 → M = 67.9, SD = 24.3 → M = 67.4, SD = 21.8; control group M = 87.3, SD = 20.8 → M = 82.6, SD = 23.2 → M = 76.4, SD = 23.8<br>**depression**<br>• non-sig. between-group effect at 6 months (data NR)<br>• within-group: intervention group M = 34.2, SD = 11.7 → M = 17.1, SD = 13.0 → M = 20.2, SD = 19.2; control group M = 35.3, SD = 13.4 → M = 26.6, SD = 15.3 → M = 23.4, SD = 16.6	National Institute for Health and Care Research – Research for Patient Benefit Programme

Heisel	men (55–70) struggling with retirement transition, but not screened positive for severe suicidal ideation or an active-untreated major mental disorder	*Meaning-Centered Men's Groups / MCMG*:<br>an existentially oriented group intervention aiming to enhance psychological resilience, consisting of 12 weekly 90-minute sessions delivered by two male facilitators (the project P.I. and a community-based social service provider). Groups of 10 men explore meaning in creativity, experiences, attitudes, life, and generativity, while developing camaraderie and peer support. Each session includes a brief reminder of the previous theme, a check-in, facilitator input, group discussions and "go-arounds", and periodic handouts and group exercises.<br>*duration: 12 weeks	*MCMG*	no control group	1.suicidal ideation: GSIS, including subscales on suicide ideation (SI), death ideation (DI), loss of personal self-worth (LOSS), and perceived meaning in life (MIL)<br>2.depression: GDS<br>*assessment: baseline, 12 weeks (post-intervention)<br><br>**Data for 6 weeks (mid-intervention), 3- and 6-month follow-up assessments after intervention completion not reported for any measures	**suicidal ideation**<br>• sig. pre–post (within-group) decrease (MD = −4.9, 95% CI [−8.3, −1.4], p = 0.008, d = 0.56), from M = 47.8, SD = 12.2 to M = 42.9, SD = 10.8<br>among four subscales<br>• sig. decrease in SI<br>• non-sig. change in DI<br>• sig. decrease in LOSS<br>• sig. increase in MIL<br>**depression**<br>• sig. pre–post (within-group) decrease (MD = −2.2, 95% CI [−4.0, −0.5], p = 0.014, d = 0.57), from M = 6.2, SD = 5.7 to M = 4.0, SD = 3.2	Movember Foundation – Men's Mental Health Grant; Canada Research Chairs Program (supporting G.L. Flett)
Jackson	men (30–64) at suicide risk, experiencing financial or other difficulties in Bristol, North Somerset, and South Gloucestershire	*Hope service*:<br>a hybrid intervention primarily featuring face-to-face motivational interviewing sessions to address acute distress and financial difficulties (number of sessions based on need). During COVID-19 lockdowns, support shifts to telephone delivery or socially distanced face-to-face formats. Supplementary support includes phone calls and text messages.<br>*duration: November 2018–October 2020	moderate/high-intensity user of *Hope service*	low-intensity user of *Hope service*	1.suicidal ideation: PHQ-9 (4-point item, dichotomized as yes/no for analysis)<br>2.depression: PHQ-9 (total score)<br>*assessment: baseline, 6 months (follow-up)	**suicidal ideation**<br>• non-sig. between-group difference (adj. OR = 0.4, 95% CI [0.1, 2.3], p = 0.31; unadj. OR = 1.0, 95% CI [0.3, 3.0], p = 1.00)<br>• 55% decrease in the proportion of service users reporting suicidal ideation in the past 2 weeks (all participants; % difference = −52.5%, 95% CI [−64.1%, −40.9%]); 56% decrease among low-intensity users (% difference = −56.3%, 95% CI [−80.6%, −31.9%]); 54% decrease among moderate/high-intensity users (% difference = −51.6%, 95% CI [−64.8%, −38.4%]) (95% CI excludes zero, suggesting a statistically significant decrease)<br>**depression**<br>• non-sig. between-group difference (adj. MD = −1.6, 95% CI [−5.1, 2.0], p = 0.38; unadj. MD = 0.7, 95% CI [−3.0, 4.3], p = 0.72)<br>• 49% decrease in mean depression scores (all participants; MD = −10.0, 95% CI [−11.7, −8.3]); 54% decrease among low-intensity users (MD = −11.3, 95% CI [−14.3, −8.4]); 48% decrease among moderate/high-intensity users (MD = −9.6, 95% CI [−11.6, −7.6]) (95% CI excludes zero, suggesting a statistically significant decrease)	National Institute for Health and Care Research – Applied Research Collaboration West; Second Step
De Leo	males (24–60) with severe suicidal ideation and/or attempts from various psychiatric diagnoses, after discharge from inpatient care at Gold Coast Hospital, Queensland	*Intensive Case Management / ICM*:<br>a post-discharge follow-up program including weekly face-to-face sessions with a community case manager and twice-weekly telephone check-ins from LifeLine counselors. Services also include Individual Program Plans, home visits, skills-building, problem-solving, etc. to enhance continuity of care, support recovery, and improve community functioning.<br>*duration: 12 months	*ICM*	TAU<br>usual post-discharge care including pharmacotherapy, referrals to general practitioners, psychologists, psychiatrists, rehabilitation services, and routine case management (not provided by *ICM* case managers), but without an Individualized Program Plan or LifeLine counselor telephone calls	1.suicide death<br>2.suicidal ideation: SSI<br>3.depression: BDI-II<br>4.help-seeking behavior – health service use: structured interview assessing contact with health services for mental/physical health needs or in response to suicidal crises, medication compliance, and attendance at outpatient appointments<br>5.help-seeking behavior – professional contacts: frequency (often/rarely) and mode (home, office, telephone) of contacts with various professionals<br>*assessment: baseline, 6 months (mid-intervention), 12 months (post-intervention)	**suicide death**<br>• none recorded in either group over the 12-month period<br>**suicidal ideation**<br>• non-sig. main effect of treatment group and non-sig. treatment × time interaction (data NR)<br>• sig. within-group decrease in intervention group from 6 months (M = 7.50, SD = 7.88) to 12 months (M = 4.43, SD = 6.84) [t(13) = 2.26, p < 0.05]; non-sig. change from baseline (M = 9.36, SD = 10.35) to 12 months [t(13) = 2.03, p = 0.064]<br>• within-group change in control group: baseline (M = 10.38, SD = 14.41) → 6 months (M = 7.63, SD = 11.07) → 12 months (M = 6.75, SD = 11.30) (significance NR)<br>**depression**<br>• non-sig. main effect of treatment group and non-sig. treatment × time interaction (data NR)<br>• sig. within-group decrease in intervention group from baseline (M = 28.21, SD = 11.27) to 6 months (M = 18.50, SD = 11.45) [t(13) = 3.82, p < 0.01] and from baseline to 12 months (M = 16.50, SD = 9.94) [t(13) = 3.94, p < 0.01]<br>• within-group change in control group: baseline (M = 27.25, SD = 18.84) → 6 months (M = 23.88, SD = 17.01) → 12 months (M = 23.13, SD = 17.90) (significance NR)<br>**help-seeking behavior – health service use**<br>• a higher proportion of participants in the intervention group reported contact with health services compared with the control group, including psychiatrists, general practitioners, psychologists, social workers, psychiatric nurses, counselors, and other health workers<br>• case managers were the most reported service: intervention group: 13/14 at 6 months and 12/14 at 12 months; control group: 5/8 and 2/8, respectively (Note: this likely reflects a process/fidelity indicator rather than an effectiveness outcome, given that case management was a core component of the intervention)<br>**help-seeking behavior – professional contacts** not reported	Commonwealth of Australia, Department of Health and Ageing, under the National Suicide Prevention Strategy

Abbreviations: AHSQ = Actual Help-Seeking Questionnaire; ASIQ = Adult Suicidal Ideation Questionnaire; ATSPPH-SF = Attitudes Toward Seeking Professional Psychological Help Scale-Short Version; BDI-II = Beck's Depression Inventory II; BSSI = Beck Scale for Suicidal Ideation; C-SSRS Screen Version = Columbia-Suicide Severity Rating Scale Screen Version; GDS = Geriatric Depression Scale; GHSQ = General Help-Seeking Questionnaire; GSIS = Geriatric Suicide Ideation Scale; HAM-D = Hamilton Depression Rating Scale; HANDS = Harvard National Depression Screening Day Scale; HSUQ = Health Services Use Questionnaire; MDRS-7 = Male Depression Risk Scale-7; PHQ-9 = Patient Health Questionnaire-9; SBQ-R = Suicide Behaviors Questionnaire-Revised; SPS = Suicide Probability Scale; SSDS = Self-Stigma of Depression Scale; SSI = Scale for Suicide Ideation; YRBS = Youth Risk Behavior Survey

sig. = statistically significant; non-sig. = statistically non-significant; p = p-value; CI = confidence interval; t = t-statistic; adj. = adjusted; unadj. = unadjusted; IPTW = inverse probability of treatment weighting; M = mean; SD = standard deviation; SE = standard error; n = number (sample size); b = unstandardized regression coefficient (slope); β = standardized regression coefficient; coef. = coefficient; OR = odds ratio; MD = mean difference; SMD = standardized mean difference; d = Cohen's d; NR = not reported; vs. = versus; n/a = not applicable; TAU = treatment as usual

Supplementary Table S3. Risk of bias justifications for non-randomized articles (ROBINS-I V2, 2024 Version)

Article	Confounding*	Classification of interventions	Selection of participants	Deviations from intended interventions (effect of assignment)	Missing outcome data	Measurement of outcome	Selection of reported result	Overall
Stas *Get out of your head*	serious no confounder control	low	serious selection into the analysis depended on completion of the post-assessment, likely related to both intervention and outcome	low	serious very severe attrition (43%); MAR assumed but not validated; MNAR cannot be ruled out (missingness may depend on true outcome value); no sensitivity analysis	moderate participants were not blinded; outcomes were self-reported; awareness of receiving the intervention may influence outcome assessment	moderate no protocol/SAP	serious
Ogrodniczuk *HeadsUpGuys*	serious no confounder control	low	serious selection into the study depended on visiting the intervention website and completing the survey with complete responses, likely related to both intervention and outcome	low	low all variables sum to n=443 (except "Knowledge of next steps" item n=442), suggesting a preselected cleaned/ complete-response analytic sample	moderate participants were not blinded; outcomes were self-reported; awareness of the intervention may influence outcome assessment	moderate no protocol/SAP	serious
Schlichthorst *Man Up*	moderate insufficient confounder control; adjusted for key sociodemographic confounders but not key baseline outcome-related confounders	serious intervention status classified based on self-reported viewing of *Man Up* in the post-screening survey (collected alongside outcomes); misclassification possible and likely differential	serious selection into Groups B/C depended on completion of the post-screening survey, and selection into Group C further depended on self-reported viewing of *Man Up*, likely related to both intervention and outcome	low	serious missing data apparent in Tables 1 and 2 when summing counts; unclear whether outcomes (Table 3) have unreported missingness; available-case analysis; missingness likely related to true outcome value; no sensitivity analysis	moderate participants were not blinded; outcomes were self-reported; knowledge of exposure status (viewed vs not viewed *Man Up*) may influence outcome assessment	moderate no protocol/SAP	serious
Daigle *SPW 2000*	serious no confounder control	serious intervention status classified based on self-reported exposure to *SPW 2000* in a cross-sectional survey (collected alongside outcomes); misclassification possible and likely differential	serious selection into exposed vs non-exposed groups depended on self-reported exposure to the campaign (i.e., noticing/remembering *SPW 2000*), likely related to both intervention and outcome	low	moderate exposed (n=190) + non-exposed (n=830) = 1020; insufficient information to judge missingness	moderate participants were not blinded; outcomes were self-reported; knowledge of exposure status (exposed vs non-exposed) may influence outcome assessment	moderate no protocol/SAP	serious
Nakao *EAP*	serious no confounder control	low	serious selection into the analysis depended on remaining in the company and attending the periodic health examination in 2005, likely related to both intervention and outcome	low	serious high attrition (31% Int, 29% Con); complete-case analysis; missingness likely related to true outcome value; no sensitivity analysis	moderate no blinding; HAM-D ratings involved observer judgement; knowledge of intervention status (*EAP* vs reference group) may influence outcome assessment	moderate no protocol/SAP	serious
Calear *Silence is Deadly*	serious inadequate confounder control; adjustment limited to a small set of participant/ school characteristics and did not include key baseline outcome-related confounders	low	serious selection into the analysis was affected by cluster loss due to poor intervention attendance and scheduling conflicts, likely related to both intervention and outcome	low	serious extremely severe and highly differential attrition (64% Int, 27% Con at 6–12 weeks follow-up), including loss of two intervention clusters; MAR assumed but not validated; MNAR cannot be ruled out (missingness may depend on true outcome value); no sensitivity analysis for missingness mechanism	moderate participants were not blinded (open-label); outcomes were self-reported; knowledge of intervention status (intervention vs waitlist control) may influence outcome assessment	low	serious
Heisel *MCMG*	serious no confounder control	low	serious selection into the analysis depended on completion of the full *MCMG* course, likely related to both intervention and outcome	moderate analysis based on participants who completed the full *MCMG* course (27/30)	moderate outcome data missing for 2/30 participants; complete-case analysis; missingness likely related to true outcome value; no sensitivity analysis	moderate participants were not blinded; outcomes were self-reported; awareness of receiving the intervention may influence outcome assessment	serious no publicly available protocol/SAP; results only reported for post-intervention (12-week), omitting mid-intervention (6-week) as well as 3- and 6-month follow-up results	serious
Jackson *Hope service*	low extensive confounder control	low	serious selection into the analysis depended on completion of the baseline questionnaire, attendance at the final session, and completion of the follow-up questionnaire, with additional selection based on *Hope* workers' judgement of appropriateness (e.g., excluding service users deemed highly vulnerable or at imminent risk of suicide), likely related to both intervention and outcome	low	moderate high attrition (24%); among completers, outcome data for suicidal ideation and depression were complete; MICE (20 datasets) used for limited covariate missingness; complete-case sensitivity analysis showed similar results; MNAR cannot be ruled out	moderate participants were not blinded; outcomes were self-reported; knowledge of exposure intensity (low vs moderate/high intensity service use) may influence outcome assessment	moderate no protocol/SAP	moderate

Supplementary Table S4. Risk of bias justifications for randomized articles (RoB 2)

Article	Randomization process	Deviations from intended interventions (effect of assignment)	Missing outcome data	Measurement of outcome	Selection of reported result	Overall
Frey<br>*Man Therapy*	low	high<br>open-label; per-protocol analysis – excluded 74 intervention participants who did not view *Man Therapy*	high<br>severe and differential attrition (39% Int, 25% Con at 12 weeks); complete-case analysis; Little's MCAR test did not reject the MCAR assumption but cannot rule out MNAR (missingness related to true outcome value)	some concerns<br>study was open-label; participants were outcome assessors; knowledge of intervention assignment may influence outcome assessment	some concerns<br>no SAP; "active participation in mental health treatment" outcome not pre-specified	high
Gilgoff<br>*Man Therapy*	low	high<br>open-label; per-protocol analysis – excluded 74 intervention participants who did not view *Man Therapy*	high<br>very severe and differential attrition (42% Int, 30% Con at 12 weeks); complete-case analysis; missingness likely related to true outcome value; no sensitivity analysis	some concerns<br>study was open-label; participants were outcome assessors; knowledge of intervention assignment may influence outcome assessment	high<br>no SAP; "professional and non-professional help-seeking behavior" outcomes not pre-specified and established for this paper; non-significant unadjusted effect (p = 0.096) became significant (p = 0.049) only after adjustment using covariates selected in a data-driven way (p < 0.10 threshold)	high
King<br>*Man Up*	low	low<br>ITT reported, but corresponds to modified ITT due to exclusion of randomized participants from analysis (lost to follow-up)	low<br>minimal participant loss; completeness of outcome data uncertain but less likely to materially affect results	some concerns<br>study claims "double-blinding" (participants blinded), but participants could likely discern intervention assignment; participants were outcome assessors; knowledge of intervention assignment may influence outcome assessment	low	low
Nicholas<br>*Boys Do Cry*	low	low<br>participants not blinded; ITT (as reported and methodologically robust)	low<br>minimal participant loss and minimal missing outcome data	some concerns<br>study was single-blinded (participants not blinded); participants were outcome assessors; knowledge of intervention assignment may influence outcome assessment	low	low
Milner 2019<br>*Contact+Connect*	low	some concerns<br>participants not blinded; contamination risk arising from the trial context due to waitlist control; modified ITT (analysis restricted to randomized participants who returned follow-up survey and were "able to be included in analysis")	high<br>severe attrition (35% overall); complete-case analysis; missingness likely related to true outcome value; no sensitivity analysis	some concerns<br>participants were not blinded and were outcome assessors; knowledge of intervention assignment may influence outcome assessment	some concerns<br>suicidal ideation pre-specified as measured using GHQ-28 in protocol and SBQ-R in registry (conflict); publication used SBQ-R; added not pre-specified outcome "communication about suicide"	some concerns
Milner 2017<br>*Contact+Connect*	low	some concerns<br>participants not blinded; contamination risk arising from the trial context due to waitlist control; modified ITT (analysis restricted to randomized participants who returned follow-up survey and were "able to be included in analysis")	high<br>severe attrition (30% overall); complete-case analysis; missingness likely related to true outcome value; no sensitivity analysis	some concerns<br>participants were not blinded and were outcome assessors; knowledge of intervention assignment may influence outcome assessment	some concerns<br>registry and protocol specified "shame", "self-blame", and "social inadequacy" subscales of SSDS, but not "help-seeking inhibition"; publication included "help-seeking inhibition" and omitted "social inadequacy"	some concerns
Jerant<br>*MAPS*	low	low<br>participants not blinded; modified ITT (excluding 6 participants who did not attend the study visit and 4 participants found to be ineligible post-randomization)	low	some concerns<br>participants were not blinded (study claimed "double-blinding: investigator/outcome assessor") and were outcome assessors; knowledge of intervention assignment may influence outcome assessment	high<br>no SAP; focused on a single pre-specified secondary outcome "discussion of suicidal thoughts", with omission of all other pre-specified outcomes (unclear if analyzed)	some concerns
Pratt<br>*CBSP*	low	some concerns<br>participants not blinded; claimed ITT, but lacked transparency due to missing analysis population in CONSORT flow diagram; likely modified ITT	high<br>very severe attrition (45% Int, 42% Con at 6 months); MAR assumed but not validated; MNAR cannot be ruled out (missingness may depend on true outcome value); no sensitivity analysis	some concerns<br>primary outcome (suicidal and self-injurious behavior) was objective (record-based) and assessed by single-blind raters; however, participants were not blinded, and participant-reported outcomes (BSSI, SPS, BDI-II) may be influenced by knowledge of intervention assignment	some concerns<br>no SAP	some concerns
De Leo<br>*ICM*	low	high<br>open-label; per-protocol analysis – only treatment-complier results were calculated under a case-complete analysis framework	high<br>extremely severe and differential attrition (53% Int, 73% Con at 12 months); LOCF attempted but not feasible; complete-case analysis used; missingness likely related to true outcome value; no sensitivity analysis	some concerns<br>study was open-label; participants were outcome assessors for most items (except SSI, intended as clinician-rated semi-structured interview); knowledge of intervention assignment may influence outcome assessment	some concerns<br>no SAP; "health service use" and "professional contacts" outcomes not pre-specified	high

***Key confounders for help-seeking:** baseline help-seeking attitudes/intentions or prior mental health service use, baseline mental health literacy, baseline mental health symptom severity, access to care/service availability, socioeconomic status (SES), and age
Key confounders for depression: baseline depression severity, concurrent treatment at baseline (e.g., psychotherapy/medication), psychiatric comorbidity, major life stressors, socioeconomic status (SES), age, sexual orientation, and race/ethnicity
Key confounders for suicidality: baseline suicidality (including prior suicide attempts), baseline depression severity, substance use disorder/alcohol or drug use, concurrent treatment at baseline (including crisis care/medication changes), major acute life stressors, socioeconomic status (SES), age, sexual orientation, and race/ethnicity

Abbreviations: ITT = intention-to-treat; PP = per-protocol; MAR = missing at random; MNAR = missing not at random; MCAR = missing completely at random; SAP = statistical analysis plan; Int = intervention group; Con = control group; LOCF = last observation carried forward; MICE = multiple imputation by chained equations

Risk-of-bias assessments were conducted using a calibrated approach across studies. Domain-level assessments were conservative, and overall judgments were informed by these assessments but were not derived through a strictly algorithmic rule, reflecting consideration of the relative importance and potential impact of biases.

Supplementary Table S5. Gender-tailoring data extracted from included interventions

Intervention	Gender-tailoring data<br>addressing male-specific barriers to help-seeking and mental health
EAP	No explicit gender-tailoring strategies described
CBSP	No explicit gender-tailoring strategies described
ICM	No explicit gender-tailoring strategies described
SPW 2000	1 *SPW 2000* promoted men's right to feel pain and seek help. The slogan, "Pain is not gender-specific – yet 80% of suicides are committed by men", aimed to change the belief that men should not feel pain or express suffering.
Hope service	1 Rising suicide rates among men have been linked to economic recession and associated financial difficulties. *Hope service* addresses acute distress among suicidal men, while also tackling debt, financial, employment, and welfare difficulties.
MAPS	1 The initial intervention version drew on theory and research on male help-seeking for psychological distress.<br>2 The final intervention version incorporated feedback from interviews with stakeholders in male suicide prevention.<br>3 The intervention aimed to promote disclosure and discussion of recent suicidal thoughts with primary care clinicians.
Get out of your head	1 The campaign was developed with input from an expert group comprising professionals who work with the target population and in mental health, as well as a focus group of the target audience.<br>2 The campaign aimed to improve men's mental health, break stigma around men's mental health, improve recognition of signs of struggle, and increase help-seeking behavior and peer support.<br>3 The website provided information tailored to men on how to recognize signs of suicidality, how to take care of oneself and others, how to start conversations about mental health problems and suicidality, and how to seek professional help and access other relevant resources.<br>4 Four video testimonials featured men who had experienced mental health struggles.
MCMG	1 Retirement may be daunting for men who derive a sense of identity from work and career, as it can lead to reductions in purposeful activity and a sense of routine, and can confer suicide risk. *MCMG* aims to enhance psychological resilience and prevent the onset or exacerbation of suicide risk among men facing this transition.<br>2 Each group comprised 10 male participants and two male facilitators: the project P.I. and a community-based social service provider.<br>3 A group format was chosen so that members could attend to and support one another, enhancing camaraderie and social support.<br>4 Group members were encouraged from the outset to stay in contact after the program ended to maintain cohesion, camaraderie, mutual support, and the benefits gained from *MCMG*.<br>5 Recruitment efforts included outreach at car shows and hosting a men's retirement and leisure fair.<br>6 *MCMG* was advertised as a "men's group dealing with adjustment to retirement", rather than a "psychotherapy group", to normalize participant concerns about retirement.
Contact+Connect	1 A focus group with 20 participants was planned to develop intervention ideas and materials, and to optimize industry/male blue-collar worker relevance and framing, preferred modality, frequency, and timing of BCIs, as well as attractiveness, clarity, and usefulness of the content.<br>2 The intervention was largely informational and educational, with content organized around three main themes: debunking myths and stereotypes, normalization, and empowerment.<br>3 The intervention included an anti-stigma component and aimed to dispel myths about mental health problems in oneself and others.<br>4 The intervention provided basic information to improve mental health literacy and targeted knowledge about risk and protective factors for depression and suicide.<br>5 The intervention provided information on help-seeking, aiming to improve knowledge about help-seeking, and included links to sources of help.<br>6 The intervention encouraged the establishment and maintenance of long-term contact with others, encouraged social connection, and aimed to increase knowledge about communication and the importance of social support through connections with friends, family, and work colleagues.
Boys Do Cry	1 The adapted lyrics highlighted how men are typically discouraged from discussing their difficulties, and how men are typically encouraged to remain stoic, keep their difficulties to themselves, and never cry.<br>2 The lyrics encouraged men to express their feelings and seek support from others when experiencing mental health difficulties or when not coping well.<br>3 The lyrics emphasized the need for men to talk to others when experiencing mental health difficulties and to encourage others to do the same.<br>4 The video ended with the call to action: "When the going gets tough. Get Talking".<br>5 The video featured a large and diverse group of men only, creating the appearance of a support group.<br>6 The study partnered with an Australian not-for-profit men's suicide prevention organization, Gotcha4Life, for recruitment.<br>7 Recruitment efforts also included targeting male-dominated university disciplines (e.g., engineering and business) and snowballing via existing participants.<br>8 The music video intervention was accompanied by a supplementary website (not live during the trial) that provided additional resources, including six short interviews with men from the music video, information on who to talk to about mental health difficulties (e.g., a men's mental health telehealth service, crisis lines, or a GP), and how to talk to someone experiencing mental health difficulties or thinking about suicide.

Silence is Deadly	1	The intervention focused on masculine norms and their impacts on health behaviors and promoted an understanding of the complex role of masculinity in the lives of boys and men, aligning with research calling for psychoeducational programs for boys in schools and APA guidelines on psychological practice with boys and men.
	2	Key statistics about mental health, suicide, and help-seeking were presented to normalize the existence of mental health issues and highlight the low help-seeking rates among men.
	3	The program discussed the explicit ways in which masculine norms can hinder early and effective help-seeking.
	4	Male role modeling and social norming were central to the program. Menslink presenters primarily delivered the presentation, with local celebrity athletes serving as guest presenters or appearing in videos to reinforce key messages.
	5	In classroom presentations, Menslink presenters and guest athletes shared their own positive help-seeking experiences (in response to personal experiences of dealing with tough times), highlighted the value of speaking to a mate, and reflected on lessons learned in challenging traditional masculine norms.
	6	In videos, athletes challenged gender norms that prevent positive help-seeking attitudes and emphasized the importance of communicating about personal issues to prevent suicide.
	7	The program emphasized both seeking and providing peer support, including strategies on how to “help a mate” (i.e., starting a conversation when a friend is not doing well or expressing suicidal thoughts).
	8	The program also encouraged seeking help from adult sources, such as parents, teachers, and health professionals.
	9	The intervention used male-tailored vocabulary, such as “mate” instead of “friend”, within the Australian context.
HeadsUpGuys	1	The intervention was developed with input from men who had experienced depression and suicidality.
	2	Targeted information, practical advice, and strategies were provided for managing and recovering from depression and suicidality, specifically tailored to a male audience to bolster men’s mental health literacy.
	3	The program aimed to reduce stigma by dismantling stigmatized beliefs about men’s mental health challenges.
	4	The program sought to norm and validate reaching out to peers and/or professionals for support.
	5	A graduated approach to help-seeking was promoted, starting with self-management and progressing to external support.
	6	Both self-management and external help-seeking were framed as embodiments of masculine strength and courage. This challenged traditional stigma around men’s vulnerability and highlighted the resilience involved in effective help-seeking.
	7	The intervention promoted hope, encouraging men to take action toward recovery.
	8	Strengths-based approaches were used, empowering and upskilling men to manage their own health while norming the inclusion of others to support their health management.
	9	The website encouraged men to share the platform with others, fostering mutual peer support. Sharing resources among men does not violate masculine norms around directly asking for help or disclosing mental health challenges.
	10	Anonymity and privacy were prioritized by the e-health platform, aligning with men’s preferences for independence, autonomy, and self-reliance.
	11	Testimonials and success stories were included to motivate users and further reduce stigma around seeking mental health support.
Man Therapy	1	The intervention aimed to help men explore how gender socialization influences relationships and mental health, and gender-specific issues that increase the risk for suicide and mental health problems, especially depression; change cultural norms to reduce stigma about mental health and suicide; empower men to seek help and promote help-seeking as a sign of strength rather than weakness; and ultimately reduce depression and suicide risk.
	2	*Man Therapy* framed its approach around hope and resilience while integrating humor.
	3	Humor was situated within a positive psychology and positive masculinity framework as a method of coping and healing for men, using male-oriented language, metaphors, and wordplay (e.g., car repair, fishing) alongside serious information.
	4	The website featured a fictional “therapist” host, Dr. Rich Mahogany, a no-nonsense “man’s man” who promoted mental health education and help-seeking and emphasized honest talk about life’s challenges.
	5	The website included online assessments to help men self-identify suicide risk and protective factors and encourage taking appropriate help-seeking responses.
	6	The website provided “manly mental health information, facts, and tips”, “man therapies”, and videos of men with lived experience.
	7	Action-oriented treatment styles were used, focusing on skills practice, review, and feedback on progress to accomplish goals.
	8	The intervention offered an anonymous, free, and easily accessible online screening and resource that did not require visits to therapists or other mental health professionals.
	9	The intervention implemented strategies (e.g., humor) in response to what men told the developers of *Man Therapy* they wanted in an online suicide prevention program.
	10	Online resources continued to expand to respond to the changing needs of working-aged men.
	11	Recruitment materials were customized through collaboration with community organizations to appeal to Michigan men.
	12	Such recruitment materials featured themes such as sports, fitness, hanging out with friends, and work, including sports-themed ads (e.g., basketball, golf) and specific messages for high-risk male occupational groups (e.g., first responders, veterans, miners).
	13	Online ads used less traditional mental health messaging, incorporating more graphics and information relatable to men.
	14	The program partnered with men who shared their stories and encouraged peers to visit the *Healthy Man Michigan* website, supporting dissemination of *Man Therapy*’s creative, humorous, and informative messages through an easily shareable online resource among men.

Man Up	1	An expert Advisory Group, comprising individuals with expertise in men's health (e.g., representatives from men's focused community and service organizations and expert consultants), provided input on the documentary and the trial design.
	2	The documentary aimed to improve men's wellbeing by reducing conformity to traditional masculine norms, such as stoicism, self-reliance, avoidance of emotional expression, "toughing it out", and coping alone, which hinder help-seeking and worsen mental health.
	3	*Man Up* examined how society shapes the way men and boys see themselves and behave, and how this can affect their mental health and potentially lead to suicidal thoughts.
	4	The intervention addressed the link between masculinity and its impact on help-seeking, men's mental health, and suicidality.
	5	Delivery was through a documentary hosted by Gus Worland, a well-known Australian male media personality.
	6	Men from all walks of life were featured in the documentary, modeling positive health behaviors such as talking about personal problems, expressing emotions, and seeking help.
	7	Gus spoke to men who had experienced mental health problems and/or had made suicide attempts.
	8	A recurring theme emphasized that "being a man" was often seen as mutually exclusive with reaching out to formal or informal sources of help.
	9	Real-life examples illustrated how reaching out for help had changed their trajectory for the better.
	10	Gus also spoke with mental health professionals and men's health experts, who reinforced the messages from "the men on the street".
	11	Gus's visit to Lifeline Australia helped demystify how crisis support services operate.
	12	The intervention was named "Man Up" to deliberately challenge the conventional use of the term (e.g., "Harden up", "Suck it up", "Man up").
	13	In Episode 1, Gus spoke with men who had experienced suicidal crises about what led them to this point and what got them through.
	14	In Episode 2, Gus spoke with individuals and organizations that are encouraging men to open up to their mates to combat suicide.
	15	In Episode 3, a campaign advertisement with the tagline "Man Up, Speak Up" was created to raise awareness of the damage caused by men "toughing it out".
	16	Recruitment efforts included outreach through sporting clubs and the networks of the Advisory Group.
